# Assessing reproducibility of Hi-C chromatin interactions using stratum-adjusted irreproducible discovery rate

**DOI:** 10.1093/bioinformatics/btag390

**Published:** 2026-08-19

**Authors:** Chen Xue, Feipeng Zhang, Qunhua Li

**Affiliations:** Department of Statistics, The Pennsylvania State University, University Park, PA 16802, United States; School of Economics and Finance, Xi’an Jiaotong University, Xi’an, Shaanxi 710049, PR China; Department of Statistics, The Pennsylvania State University, University Park, PA 16802, United States

## Abstract

**Motivation:**

Hi-C is a powerful technology for mapping chromatin interactions genome-wide. However, interaction loops identified from Hi-C contact maps often vary across replicate experiments due to experimental noise, making reproducibility assessment essential. A major challenge lies in the genomic distance dependence of interaction strength, which systematically affects reproducibility but is overlooked by existing methods for reproducibility assessment.

**Results:**

We introduce Stratum-Adjusted Irreproducible Discovery Rate (SIDR), a novel statistical model that integrates distance stratification into the widely-used Irreproducible Discovery Rate (IDR) framework. SIDR explicitly models the confounding effect of genomic distance, enabling global control of irreproducibility across interaction ranges. Through simulations and real Hi-C datasets, we demonstrate that SIDR improves discriminative power and recovers more biologically meaningful interactions than existing approaches, making it a valuable tool for robust and reproducible Hi-C analysis.

**Availability:**

The R package SIDR is freely available on GitHub https://github.com/qunhualilab/SIDR.

## 1 Introduction

Hi-C is a high-throughput genomic technology that captures the three-dimensional architecture of the genome by quantifying interaction frequencies between genomic loci in spatial proximity (Lieberman-Aiden *et al.* 2009). These interactions offer critical insights into gene regulation by revealing spatial relationships among promoters, enhancers, and insulator elements such as CTCF (Shen *et al.* 2012, Doyle *et al.* 2014, Rao *et al.* 2014).

To identify statistically significant interactions from Hi-C data, several methods have been developed, including Fit-Hi-C (Ay *et al.* 2014) and HiC-DC (Carty *et al.* 2017), along with their refinements FitHiC2 (Kaul *et al.* 2020) and HiC-DC+ (Sahin *et al.* 2021). These methods model contact frequency as a function of genomic distance and other covariates, and assess significance by comparing observed read counts to expected backgrounds. However, Hi-C data remain noisy and heterogeneous (Sefer *et al*. 2016, Sefer 2021,  2022), so even effective peak-calling methods can still yield spurious interaction calls.

When replicate Hi-C datasets are available, reproducibility analysis across replicates provides an important safeguard against false discoveries. For individual interaction calls, the Irreproducible Discovery Rate (IDR) framework (Li *et al.* 2011) is widely used in one-dimensional high-throughput assays, and its two-dimensional extension IDR2D (Krismer *et al.* 2020) adapts this idea to Hi-C by pairing overlapping interactions across replicates. However, both IDR and IDR2D assume homogeneous signal distributions and independence among irreproducible signals, assumptions that are often violated in Hi-C data. By contrast, several methods, including HiCRep (Yang *et al.* 2017), HiC-Spector (Yan *et al.* 2017), and GenomeDISCO (Ursu *et al.* 2018), assess reproducibility at the level of the entire contact map. While useful for evaluating overall dataset quality, these approaches do not provide reproducibility scores for individual interactions identified by peak-calling methods. In this work, we focus on this interaction-level reproducibility problem.

**Figure 1:**
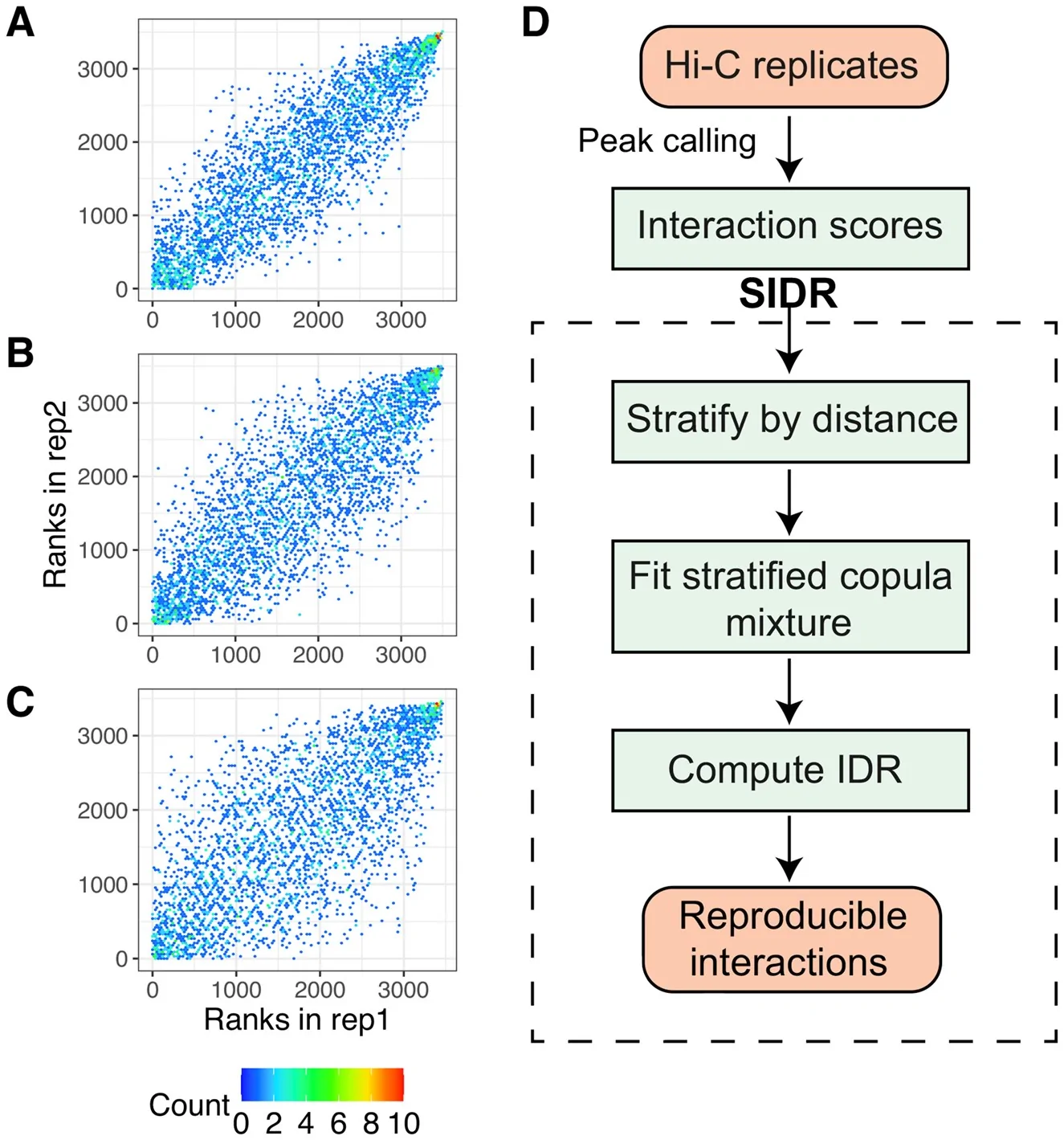
Distance dependence of replicate concordance in Hi-C interactions (A–C) and overview of SIDR (D). (A–C) Rank scatterplots of FitHiC2 −log(P-value) score for Hi-C interactions on chromosome 18 in mouse embryonic stem cells (mESCs) across a pair of replicates, stratified by genomic distance: (A) 100–200 kb, (B) 400–500 kb, and (C) 900–1000 kb. Rank correlation decreases as genomic distance increases. (D) Schematic of the SIDR procedure.

A well-documented feature of Hi-C data is that interaction frequency declines with increasing genomic distance due to the polymer nature of chromatin, which brings nearby loci into contact more frequently than distal ones (Lieberman-Aiden *et al.* 2009, Sexton *et al.* 2012). However, less well appreciated is that the *consistency* of interaction signals across replicates also declines with distance: long-range interactions are not only weaker on average but also more variable across replicates, as weaker signals are more susceptible to random noise. Figs. 1A-C illustrates this trend using FitHiC2-derived signals from mouse embryonic stem cell (mESC) data (Dixon *et al.* 2012): the correlation of significance scores across replicates declines markedly with genomic distance. This distance-dependent heterogeneity violates the assumptions of exchangeability and homogeneity underlying IDR and IDR2D, highlighting the need for a distance-aware framework.

To address these challenges, we introduce Stratum-Adjusted Irreproducible Discovery Rate (SIDR), a stratified extension of the IDR framework. SIDR partitions candidate interactions into strata based on genomic distance and estimates reproducibility parameters within each stratum. This design enables the model to accommodate structured heterogeneity in signal strength and replicate consistency, while preserving the interpretability and empirical Bayes rationale of the original IDR approach.

In the following sections, we describe the SIDR model and evaluate its performance through simulation studies and real Hi-C data analyses, demonstrating its advantages over existing methods in both statistical accuracy and biological relevance.

## 2 Methods

### 2.1 Overview of SIDR

The Stratum-Adjusted Irreproducible Discovery Rate (SIDR) is a statistical framework for assessing reproducibility of chromatin interactions in Hi-C experiments. To address the distance dependence of Hi-C signal strength and replicate concordance, SIDR stratifies candidate interactions by genomic distance and models reproducibility separately within each stratum. Within each stratum, interactions are modeled as a mixture of reproducible and irreproducible groups, following the core idea of the IDR framework but with stratum-specific parameters to capture distance-related heterogeneity. Replicate concordance within each group is described using a bivariate Gaussian distribution. The stratum-specific models are then combined to yield reproducibility scores for individual interactions while maintaining global control of the irreproducible discovery rate. A schematic overview of the SIDR workflow is shown in Fig. 1D.

SIDR therefore extends the original IDR (Li *et al.* 2011) to Hi-C data, overcoming its limitations in this setting. Unlike IDR and its two-dimensional extension IDR2D (Krismer *et al.* 2020), which assume a single homogeneous dependence structure across all signals, SIDR explicitly accounts for distance-dependent variability and background correlation. This stratified design makes SIDR particularly well-suited for Hi-C reproducibility analysis.

### 2.2 SIDR model formulation

Consider Hi-C experiments with two replicates, where $\left(y_{i,1},y_{i,2}\right)$ denote the significance scores and $d_{i}$ denotes the genomic distance for bin pair *i*. To account for the strong dependence of signal properties on genomic distance, we stratify bin pairs into *K* distance-based strata: $\left(Y_{1}^{k},Y_{2}^{k})=\{(y_{i,1},y_{i,2}):a_{k-1}<d_{i}\leq a_{k}\right\}$, with k=1,…K, where $a_{0},\ldots ,a_{K}$ are pre-specified distance thresholds. Each stratum *k* contains $n_{k}$ bin pairs, indexed by $\left(y_{j,1}^{k},y_{j,2}^{k}\right)$, for $j=1,\ldots ,n_{k}$.

Within each stratum, we assume that interactions arise from a mixture of two latent groups: reproducible (genuine) signals, which are biologically meaningful and consistent across replicates, and irreproducible (spurious) signals, which reflect noise or artifacts. We use $C_{j}^{k}$ to denote the group membership of the $j^{\text{th}}$ interaction in stratum *k*, where $C_{j}^{k}=1$ indicates reproducible and $C_{j}^{k}=0$ indicates irreproducible. The replicate dependence structure is modeled through latent variables $Z_{j}^{k}=(Z_{j,1}^{k},Z_{j,2}^{k})$, assumed to follow a stratum-specific bivariate Gaussian mixture, with realizations denoted by $z_{j}^{k}=(z_{j,1}^{k},z_{j,2}^{k})$:


$${\left(Z_{j,1}^{k},Z_{j,2}^{k}\right)}^{T}∼(1-\pi_{k})N(0,\Sigma_{0})+\pi_{k}N({\left(\mu_{k},\mu_{k}\right)}^{T},\Sigma_{1}^{k}),$$

where:

-
$\pi_{k}$ : proportion of reproducible interactions in stratum *k*.
-
$\mu_{k}>0$ : positive mean signal strength of reproducible interactions in stratum *k*, reflecting higher scores than the irreproducible interactions, which has zero mean.
-
$\Sigma_{0}=\left(\begin{array}{cc} 1 & \rho_{0}\\ \rho_{0} & 1 \end{array}\right)$ : covariance matrix for irreproducible signals, with $\rho_{0}>0$.
-
$\Sigma_{1}^{k}=\left(\begin{array}{cc} \sigma^{2} & \rho_{1}^{k}\sigma^{2}\\ \rho_{1}^{k}\sigma^{2} & \sigma^{2} \end{array}\right)$ : covariance matrix for reproducible signals in stratum *k*, with $\rho_{1}^{k}>\rho_{0}$.
-
$\sigma^{2}$ : constant variance of reproducible group.

This formulation reflects key empirical patterns in Hi-C data evident in Fig. 1A–C:

- Distance-dependent reproducibility: Short-range interactions exhibit stronger signal and better replicate concordance, while long-range interactions are weaker and more variable. To capture this structured heterogeneity, we allow both the reproducible proportion $\pi_{k}$ and mean signal strength $\mu_{k}$ to vary by stratum.
- Positive background correlation: As shown in Fig. 1A–C, even low-signal interactions exhibit nonzero correlation across replicates. We therefore model the irreproducible component with a positive correlation parameter $\rho_{0}>0$, shared across strata to provide a common baseline across genomic distances. This shared specification also improves identifiability and avoids over-parameterizing a low-signal component whose dependence pattern appears to vary much less across strata than that of the reproducible component.
- Stratum-specific replicate concordance: Fig. 1A–C shows that even among high-confidence signals, replicate concordance declines with distance. To accommodate this, the correlation $\rho_{1}^{k}$ of reproducible signals is allowed to vary across strata, with $\rho_{1}^{k}>\rho_{0}$ and potentially decreasing with *k*.
- Identifiability and parsimony: The irreproducible component variance is fixed at 1 as in IDR, and the reproducible component variance is set to a constant $\sigma^{2}$ across strata. This avoids over-parameterization while maintaining flexibility.

Because the observed Hi-C significance scores $\left(y_{j,1}^{k},y_{j,2}^{k}\right)$ are not Gaussian, we link them to the latent Gaussian variables $\left(z_{j,1}^{k},z_{j,2}^{k}\right)$ through an inverse probability transformation. This preserves the rank structure of the observed data while allowing their dependence to be modeled with a Gaussian copula, ensuring that the latent variables capture replicate concordance while the marginals match the observed distributions. Specifically, define


$$u_{j,l}^{k}=F_{k}(z_{j,l}^{k})=(1-\pi_{k})\Phi (z_{j,l}^{k};0,1)+\pi_{k}\Phi (z_{j,l}^{k};\mu_{k},\sigma^{2}),\;l=1,2,$$

where $\Phi (\cdot ;\mu ,\sigma^{2})$ is the univariate Gaussian CDF. The observed values are then:


$$y_{j,l}^{k}={\left(G_{l}^{k}\right)}^{-1}(u_{j,l}^{k}),$$

with $G_{1}^{k}$ and $G_{2}^{k}$ denoting the marginal CDFs of the observed scores in each replicate.

Let $\theta =(\pi_{k},\mu_{k},\rho_{0},\rho_{1}^{k},\sigma ),$ the likelihood of $\left(y_{j,1}^{k},y_{j,2}^{k}\right)$ is:


$$L\left(\theta \right)=\prod_{k=1}^{K}\prod_{j=1}^{n_{k}}\left[\left(1-\pi_{k}\right)\phi_{2}\left(F_{k}^{-1}\left(G_{1}^{k}\left(y_{j,1}^{k}\right)\right),F_{k}^{-1}\left(G_{2}^{k}\left(y_{j,2}^{k}\right)\right);\mathrm{0,}\Sigma_{0}\right)+\pi_{k}\phi_{2}\left(F_{k}^{-1}\left(G_{1}^{k}\left(y_{j,1}^{k}\right)\right),F_{k}^{-1}\left(G_{2}^{k}\left(y_{j,2}^{k}\right)\right);{\left(\mu_{k},\;\mu_{k}\right)}^{T},\Sigma_{1}^{k}\right)\right]/\left[f_{k}\left(F_{k}^{-1}\left(G_{1}^{k}\left(y_{j,1}^{k}\right)\right)\right)\cdot f_{k}\left(F_{k}^{-1}\left(G_{2}^{k}\left(y_{j,2}^{k}\right)\right)\right)\right],$$

where $\phi_{2}$ is the bivariate Gaussian PDF and $f_{k}$ is the PDF of the stratum-specific mixture $F_{k}$.

The SIDR framework can be generalized to cases with more than two replicates by modeling the dependence structure using a multivariate Gaussian mixture model. Details of this extension are described in Supplementary Materials Section 1.

### 2.3 Parameter estimation

We estimate the model parameters θ using the maximum likelihood procedure similar to Bilgrau *et al.* (2016). The method first approximates the marginal distributions $G_{l}^{k}$  (l=1,2) empirically, and then applies numerical optimization to maximize the log-likelihood. The procedure consists of the following steps:

1. For each stratum *k*, compute the empirical marginal CDF ${\hat{G}}_{l}^{k}(y_{j,l}^{k})=\frac{r_{j,l}^{k}}{n_{k}}$, where $r_{j,l}^{k}$ is the rank of $y_{j,l}^{k}$ and $n_{k}$ is the number of bin pairs.
2. Rescale by a factor of $\frac{n_{k}}{n_{k}+1}$ to avoid boundary issues, that is, $u_{j,l}^{k}=\frac{n_{k}}{n_{k}+1}{\hat{G}}_{l}^{k}(y_{j,l}^{k})$.
3. Initialize the parameters at $\theta =\theta_{0}$.
4. Compute pseudo-data $z_{j,l}^{k}=F_{k}^{-1}(u_{j,l}^{k}|\theta )$. Since $F_{k}^{-1}$ has no closed form, approximate it by evaluating $F_{k}$ on a grid of 1000 points for $u\in [\min(-3,\mu_{k}-3),\max(3,\mu_{k}+3)]$ and obtain $z_{j,l}^{k}$ via linear interpolation.
5. Maximize the log-likelihood of the pseudo-data using the Nelder-Mead method (Nelder and Mead 1965) to obtain $\hat{\theta }$.

### 2.4 Irreproducible discovery rate

Following the IDR and IDR2D frameworks, we quantify reproducibility of Hi-C interactions by estimating the posterior probability that a candidate belongs to the irreproducible group. For each individual interaction, reproducibility is assessed through the local irreproducible discovery rate (idr), which represents the posterior probability that the interaction is irreproducible given its observed significance scores.

Formally, the idr for the $j^{\text{th}}$ interaction in stratum *k* is


$$\text{idr}(y_{j,1}^{k},y_{j,2}^{k})=\frac{\left(1-\pi_{k})h_{0}(F_{k}^{-1}(G_{1}^{k}(y_{j,1}^{k})),F_{k}^{-1}(G_{2}^{k}(y_{j,2}^{k}))\right)}{\left(1-\pi_{k})h_{0}(F_{k}^{-1}(G_{1}^{k}(y_{j,1}^{k})),F_{k}^{-1}(G_{2}^{k}(y_{j,2}^{k})))+\pi_{k}h_{1}^{k}(F_{k}^{-1}(G_{1}^{k}(y_{j,1}^{k})),F_{k}^{-1}(G_{2}^{k}(y_{j,2}^{k}))\right)},$$

where the densities $h_{0}$ and $h_{1}^{k}$ correspond to irreproducible and reproducible groups, respectively:


$$h_{0}∼N\left(\left(\begin{array}{c} 0\\ 0 \end{array}\right),\left(\begin{array}{cc} 1 & \rho_{0}\\ \rho_{0} & 1 \end{array}\right)\right),\;h_{1}^{k}∼N\left(\left(\begin{array}{c} \mu_{k}\\ \mu_{k} \end{array}\right),\left(\begin{array}{cc} \sigma^{2} & \rho_{1}^{k}\sigma^{2}\\ \rho_{1}^{k}\sigma^{2} & \sigma^{2} \end{array}\right)\right).$$

At the dataset level, reproducibility is summarized by the global irreproducible discovery rate (IDR), which gives the expected proportion of irreproducible interactions among all discoveries below a chosen idr threshold γ:


$$\begin{array}{c} \text{IDR}(\gamma )=P(\text{irreproducible}|j\in \overset{K}{\underset{k=1}{\cup }}I_{\gamma }^{k})\\ =\sum_{k=1}^{K}\frac{\int_{I_{\gamma }^{k}}\left(1-\pi_{k}\right)dH_{0}(F_{k}^{-1}(G_{1}^{k}(y_{j,1}^{k})),F_{k}^{-1}(G_{2}^{k}(y_{j,2}^{k})))}{\int_{I_{\gamma }^{k}}dH^{k}(F_{k}^{-1}(G_{1}^{k}(y_{j,1}^{k})),F_{k}^{-1}(G_{2}^{k}(y_{j,2}^{k})))}, \end{array}$$

where $I_{\gamma }^{k}=\{(y_{j,1}^{k},y_{j,2}^{k}):\text{idr}(y_{j,1}^{k},y_{j,2}^{k})<\gamma \}$, and $H_{0}$ and $H^{k}$ are the CDFs of $h_{0}$ and $h^{k}=(1-\pi_{k})h_{0}+\pi_{k}h_{1}^{k}$, respectively. Intuitively, the local idr acts like a per-interaction reproducibility score, while the global IDR provides an overall error rate among selected discoveries.

### 2.5 Stratification and initialization

We partition interactions into distance-defined strata so as to capture changes in replicate dependence across genomic distance. The number of strata, *K*, is selected by a data-driven procedure that increases *K* until adjacent strata no longer show sufficiently distinct rank-correlation structures. Model parameters are then initialized using simple, structured heuristics that reflect expected trends across strata, with several key quantities estimated empirically to promote stable and efficient optimization. Additional details are provided in Supplementary Materials Section 3.

## 3 Results

### 3.1 Simulation setup

To evaluate the performance of SIDR and determine how to choose the number of strata *K*, we conducted three simulation with true K=3,4,5. For all simulations, the number of interaction bin pairs was fixed at 10 000. Each simulated bin pair i=1,…,10 000 was assigned a genomic distance $d_{i}$, drawn independently from an Exponential distribution:


$$d_{i}∼\text{Exponential}(0.25).$$

The exponential distribution was chosen because it captures a key property of Hi-C data: the number of bin pairs decays with increasing genomic distance, mimicking the decay in contact frequency with distance observed in real data.

To generate realistic simulation strata, we used mouse embryonic stem cell Hi-C data (50 kb resolution) from Dixon *et al.* (2012). We applied HiC-DC+ to obtain *P*-values for each bin pair, then computed the Spearman correlation of −log(p) between replicates at each genomic distance. These correlations were partitioned into *K* equal-length intervals, and the proportion of bin pairs within each interval was used to set distance thresholds in the simulated data, ensuring strata matched real-data proportions.

For each simulation, we evaluated SIDR under multiple stratum choices (K=3−5) to assess its robustness. Detailed results for K=4 are presented here, with additional results provided in the supplementary information. Based on this procedure, the strata for K=4 setting are $D_{1}=(0,0.91],\;D_{2}=(0.91,2.43],\;D_{3}=(2.43,4.65],\;D_{4}=4.65+$. These cutoffs capture short-, intermediate-, and long-range interactions, reflecting biologically distinct regimes of contact frequency and reproducibility observed in Hi-C data.

Within each stratum *k*, we simulated replicate pairs of *z*-statistics, $z_{j,l}^{k}(l=1,2)$, from a two-component Gaussian mixture (details in Supplementary Materials Section 4.1). Each *z*-statistic was converted to a one-sided *P*-value under $H_{0}:\mu_{k}=0$ vs $H_{1}:\mu_{k}>0$ via $p_{j,l}^{k}=1-\Phi (z_{j,l}^{k})$, where Φ is the standard normal CDF. These *P*-values were used as the significance scores for each bin pair.

Competitive methods: Because *P*-values are used as input, SIDR can also be viewed as a *P*-value combination framework, integrating replicate information to rank signals by consensus. To benchmark performance, we compared SIDR against both a reproducibility-based approach (IDR2D) and established *P*-value combination methods, including Fisher’s (Fisher 1925), Stouffer’s (Stouffer *et al.* 1949), and the Cauchy combination test (Liu and Xie 2020). For the *P*-value combination methods, paired *P*-values across replicates were merged and then adjusted for FDR to obtain *q*-values, while IDR2D and SIDR used −log(P-value) as input to estimate idr values. This setup allows us to directly test whether SIDR’s distance-aware stratification yields advantages over standard approaches, which either ignore replicate dependence (meta-analysis methods) or assume a homogeneous dependence structure (IDR2D).

We additionally conducted a simulation study to assess whether SIDR overfits when no distance-dependent structure is present. In this setting, all significance scores were generated from a single bivariate Gaussian mixture, with common parameters shared across all observations and no dependence on genomic distance. We then applied SIDR with K=3,4,5 and compared its performance with IDR2D and the competing *P*-value combination methods. Details are provided in Supplementary Materials Section 4.2.

### 3.2 Simulation results

**Figure 2:**
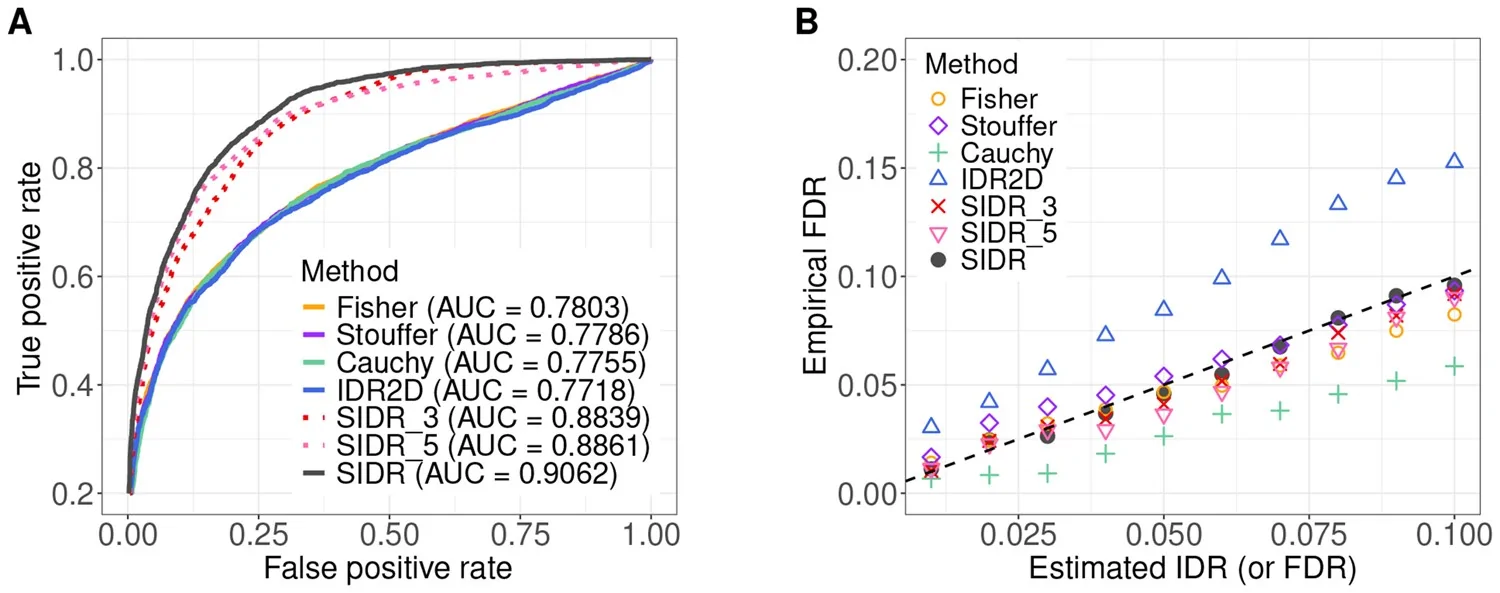
Comparison of Fisher’s method, Stouffer’s method, the Cauchy combination test, IDR2D, SIDR in simulations. The true number of strata (K=4). SIDR is shown with the true number (SIDR) and the alternative numbers of strata (SIDR_3 for K=3 and SIDR_5 for K=5). Results are evaluated based on: (A) The proportion of true positives versus the proportion of false positives; (B) The estimated error rate versus the empirical error rate.

*Discriminative power:* We evaluated performance using receiver operating characteristic (ROC) curves. For SIDR and IDR2D, ROC curves were generated by thresholding idr values, whereas for the *P*-value combination methods, they were generated by thresholding the combined *P*-values. When distance dependence was present, SIDR consistently achieved the highest true positive rate (TPR) at any fixed false positive rate (FPR), demonstrating superior discriminative power (Fig. 2A).

*Calibration:* We next assessed calibration by comparing empirical false discovery rate (FDR) with estimated significance measures: IDR for SIDR and IDR2D, and FDR for the *P*-value combination methods. As shown in Fig. 2B, SIDR maintained accurate calibration, whereas IDR2D tended to overestimate FDR and the Cauchy combination test underestimated it.

*Robustness to the choice of K:* When the true number of strata was used, SIDR recovered the model parameters with good accuracy (Supplementary Table S1). To evaluate robustness to misspecification of *K*, we also applied SIDR with K=3 and K=5 when the data were generated with K=4. This led to only minor reductions in AUC, while performance remained substantially better than that of the competing methods. Similarly, misspecification of *K* caused only mild FDR underestimation, indicating that SIDR is robust to the choices of *K*.

*Performance when distance dependence is absent:* We further considered a simulation setting in which no distance-dependent structure was present. In this case, SIDR remained competitive with the non-stratified methods and showed similar ROC performance relative to IDR2D and the *P*-value combination methods (Supplementary Fig. S3). This result indicates that the stratified framework does not overfit when stratification is unnecessary.

In summary, when distance dependence is present, SIDR consistently outperforms IDR2D and all *P*-value combination methods in discriminative power, maintains accurate calibration, and is robust to the choice of the number of strata. When no distance dependence is present, SIDR performs comparably to competing methods, indicating that it does not introduce bias when stratification is unnecessary.

### 3.3 SIDR shows strong enrichment for CTCF and cohesin in IMR90

We next evaluated SIDR on the IMR90 Hi-C dataset (Dixon *et al.* 2012), comparing it against HiC-DC+ scores from individual replicates, Fisher’s method, Stouffer’s method, the Cauchy combination test, and IDR2D on chromosome 18 at 50 kb resolution. Only interactions with genomic distances within 3 Mb were considered. To assess robustness across peak-calling pipelines, we also repeated the analysis using FitHiC2-derived peaks, with results shown in Supplementary Figs. S4 and S5. K=5 was used for all analyses.

To assess biological relevance, we evaluated enrichment of CCCTC-binding factor (CTCF) and cohesin components (RAD21, SMC3), which are well-established mediators of chromatin looping (Splinter *et al.* 2006, Hou *et al.* 2008). ChIP-seq data for CTCF, RAD21, and SMC3 were obtained from ENCODE (Feingold *et al.* 2004). Because different methods report varying numbers of significant interactions, we compared enrichment across the top n=1000, 2000, 3000, 4000, 5000 ranked interactions per method. Enrichment was defined as the percentage of interactions overlapping at least one CTCF, RAD21, or SMC3 peak.

**Figure 3:**
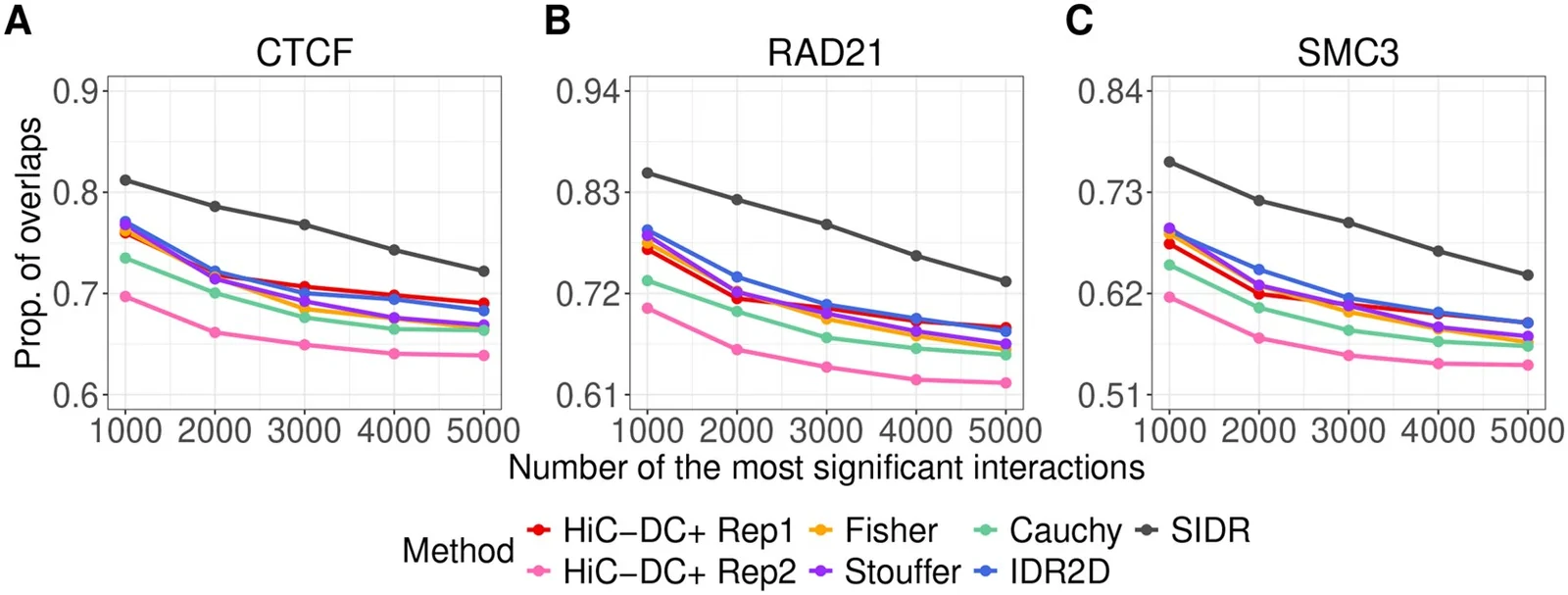
Proportions of the top 1000, 2000, 3000, 4000, and 5000 interactions identified by HiC-DC+, Fisher’s method, Stouffer’s method, the Cauchy combination test, IDR2D, and SIDR, which overlap (A) CTCF signals, (B) RAD21 signals, and (C) SMC3 signals, for chromosome 18 of the IMR90 Hi-C data at 50 kb resolution.

As shown in Fig. 3, enrichment decreased with larger *n* across all methods. Nevertheless, SIDR consistently achieved the highest enrichment for CTCF, RAD21, and SMC3 across all *n’*s. These findings demonstrate that SIDR more effectively prioritizes biologically meaningful interactions than competing approaches. Similar findings were observed in the FitHiC2-based analyses (Supplementary Fig. S4).

### 3.4 SIDR prioritizes interactions enriched for regulatory chromatin states in IMR90

Epigenetic states play a critical role in gene regulation (Hardison 2012). We next tested whether significant interactions identified by each method were enriched for ChromHMM annotations (Ernst and Kellis 2012). Using the 25-state ChromHMM segmentation for IMR90, we focused on states that are most directly linked to regulatory activity and chromatin interactions: DNase, active transcription start site (TssA), promoter upstream/downstream TSS (Prom), active enhancer (EnhA), weak enhancer (EnhWk), strong transcription (Tx), weak transcription (TxWk), and transcribed promoter/enhancer (TxEnh/Prom). DNase state marks open chromatin accessible to regulators; TssA and Prom states represent promoter-proximal regions critical for transcription initiation; EnhA and EnhWk states capture active and weak enhancers mediating long-range regulation; Tx and TxWk states indicate actively transcribed regions correlated with accessibility; and TxEnh/Prom state combines enhancer and promoter features with versatile regulatory roles. These states represent functional genomic elements most likely to participate in chromatin looping, making them appropriate for evaluating enrichment of SIDR-identified interactions. We quantified enrichment as the average number of states per bin pair among the top 5000 interactions.

**Figure 4:**
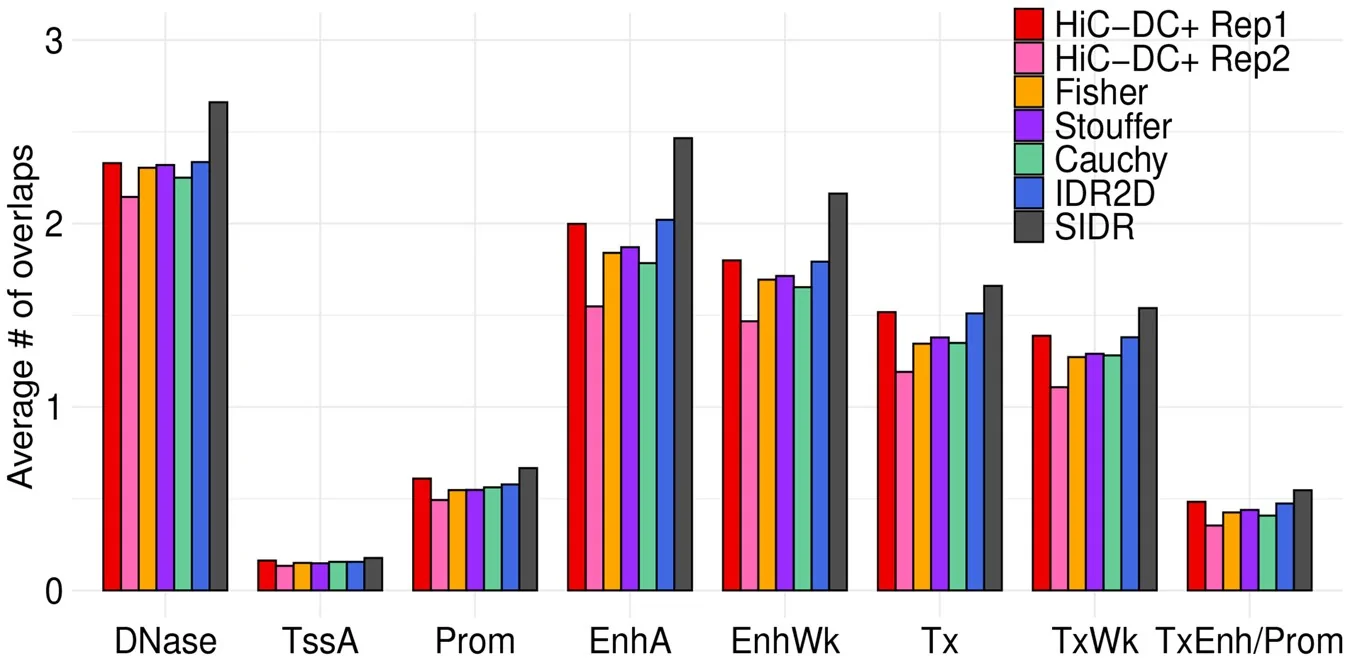
Average numbers of epigenetic states overlapping the top 5000 interactions identified by HiC-DC+, Fisher’s method, Stouffer’s method, the Cauchy combination test, IDR2D, and SIDR, for chromosome 18 of the IMR90 Hi-C data at 50 kb resolution.

As shown in Fig. 4, SIDR achieved the strongest enrichment for all ChromHMM states related to regulatory activity and chromatin interactions. This indicates that SIDR is particularly effective at prioritizing interactions anchored in promoters and enhancers, while also capturing interactions associated with open and actively transcribed regions. Consistent patterns were observed when applying SIDR to FitHiC2 scores (Supplementary Fig. S5), where it showed strongest enrichment for enhancer- and DNase-associated states and remained competitive across other annotations.

### 3.5 SIDR effectively recovers RNAPII-mediated interactions in mESC

We next evaluated SIDR on chromosome 18 of the mESC Hi-C dataset at 50 kb resolution (Dixon *et al.* 2012). Candidate interactions with genomic distances within 3 Mb were identified using both FitHiC2 and HiC-DC+, and replicate information was integrated using SIDR, Fisher’s method, Stouffer’s method, the Cauchy combination test, and IDR2D. Here we present the results based on FitHiC2, with parallel analysis using HiC-DC+ provided in the Supplementary Materials, illustrating SIDR’s robustness across different peak-calling pipelines.

**Figure 5:**
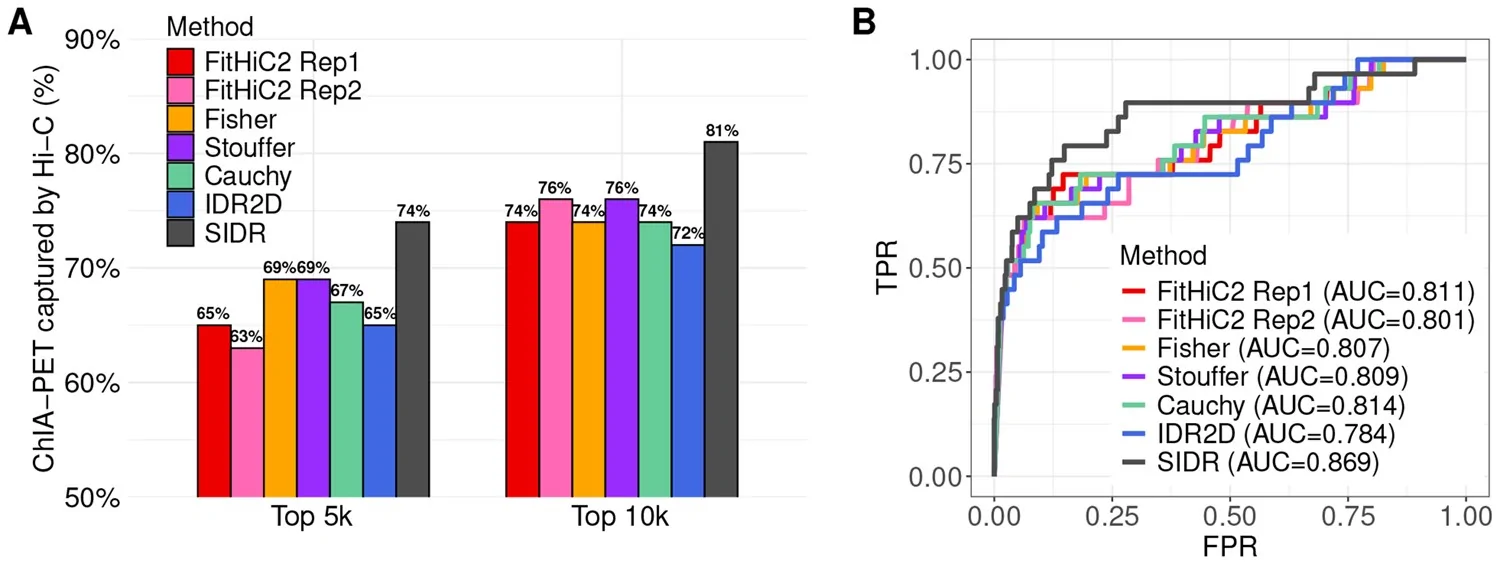
Validation of interactions identified on chromosome 18 of the mESC Hi-C data at 50 kb resolution using mESC ChIA-PET data. (A) Percentages of RNAPII ChIA-PET interactions captured by the top 5000 and 10 000 Hi-C interactions identified by FitHiC2, Fisher’s method, Stouffer’s method, the Cauchy combination test, IDR2D, and SIDR. (B) ROC curves for identifying interactions annotated by both RNAPII ChIA-PET data and RNAPII ChIP-seq data.

For validation, we used RNAPII-mediated chromatin interactions from mESC ChIA-PET experiments (Zhang *et al.* 2013), an orthogonal assay that directly detects long-range chromatin interactions mediated by specific proteins such as RNA polymerase II (RNAPII) (Fullwood and Ruan 2009). These interactions are biologically meaningful because they are strongly associated with transcriptional activity and often involve enhancer-promoter connections. We defined a ChIA-PET interaction as overlapping a Hi-C interaction if both bin pairs intersect. Among the top 5000 and 10 000 Hi-C interactions identified by each method, SIDR consistently recovered the largest number of ChIA-PET-supported interactions (Fig. 5A).

To further assess specificity, we incorporated RNAPII ChIP-seq data (Feingold *et al.* 2004). We treated ChIA-PET interactions as positives, and randomly selected an equal number of RNAPII-binding site pairs not supported by ChIA-PET as negatives. ROC analysis (Fig. 5B) showed that SIDR achieved the highest area under the curve (AUC), confirming its effectiveness in prioritizing RNAPII-mediated chromatin interactions. These observations were further supported by the HiC-DC+-based analyses (Supplementary Fig. S7).

### 3.6 SIDR captures key regulatory interaction types in mESC

To further validate biological relevance, we assessed the enrichment of CTCF-, promoter-, and enhancer-mediated chromatin interactions among significant calls. Specifically, we classified interactions into four categories based on ChIP-seq and epigenomic annotations: CTCF–CTCF, enhancer–enhancer (E–E), promoter–promoter (P–P), and enhancer–promoter (E–P). An interaction was labeled as CTCF–CTCF if both bins overlapped CTCF ChIP-seq peaks (Feingold *et al.* 2004). Similarly, E–P interactions were defined as bin pairs with one bin overlapped an active enhancer and the other a promoter, based on Bogu *et al.* (2015), with analogous criteria for E–E and P–P pairs.

**Figure 6:**
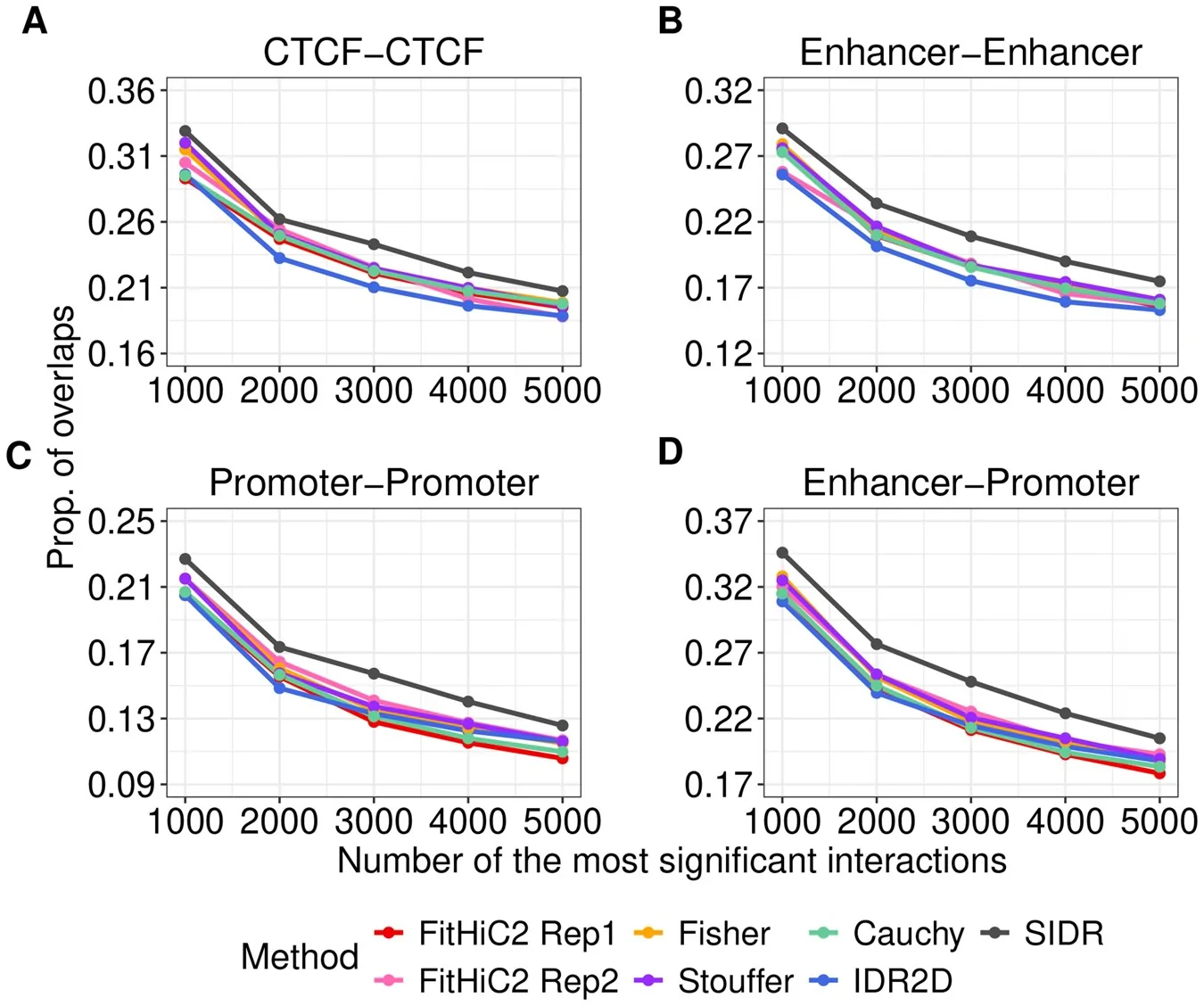
Proportions of (A) CTCF–CTCF, (B) Enhancer–Enhancer, (C) Promoter–Promoter, and (D) Enhancer–Promoter interactions among the top 1000, 2000, 3000, 4000, and 5000 Hi-C interactions identified by FitHiC2, Fisher’s method, Stouffer’s method, the Cauchy combination test, IDR2D, and SIDR, for chromosome 18 of the mESC Hi-C data at 50 kb resolution.

For each method, we evaluated the top n=1000, 2000, 3000, 4000, 5000 interactions. As shown in Fig. 6, SIDR consistently identified the highest proportions of all four functional interaction types across all *n*, demonstrating its effectiveness in prioritizing interactions anchored at regulatory elements and CTCF sites that are most likely to be biologically meaningful. The HiC-DC+-based analyses yielded similar patterns (Supplementary Fig. S8).

### 3.7 SIDR identifies interactions aligned with TAD architecture in mESC

Topologically associating domains (TADs) are genomic regions enriched for regulatory features such as epigenetic marks, cohesin complexes, and active genes (Dixon *et al.* 2012, Nora *et al.* 2012, Sexton *et al.* 2012, An *et al.* 2019). Because chromatin interactions within TADs are often enriched for functional regulatory contacts, TAD overlap provides a useful benchmark for assessing the biological relevance of interaction-calling methods.

**Figure 7:**
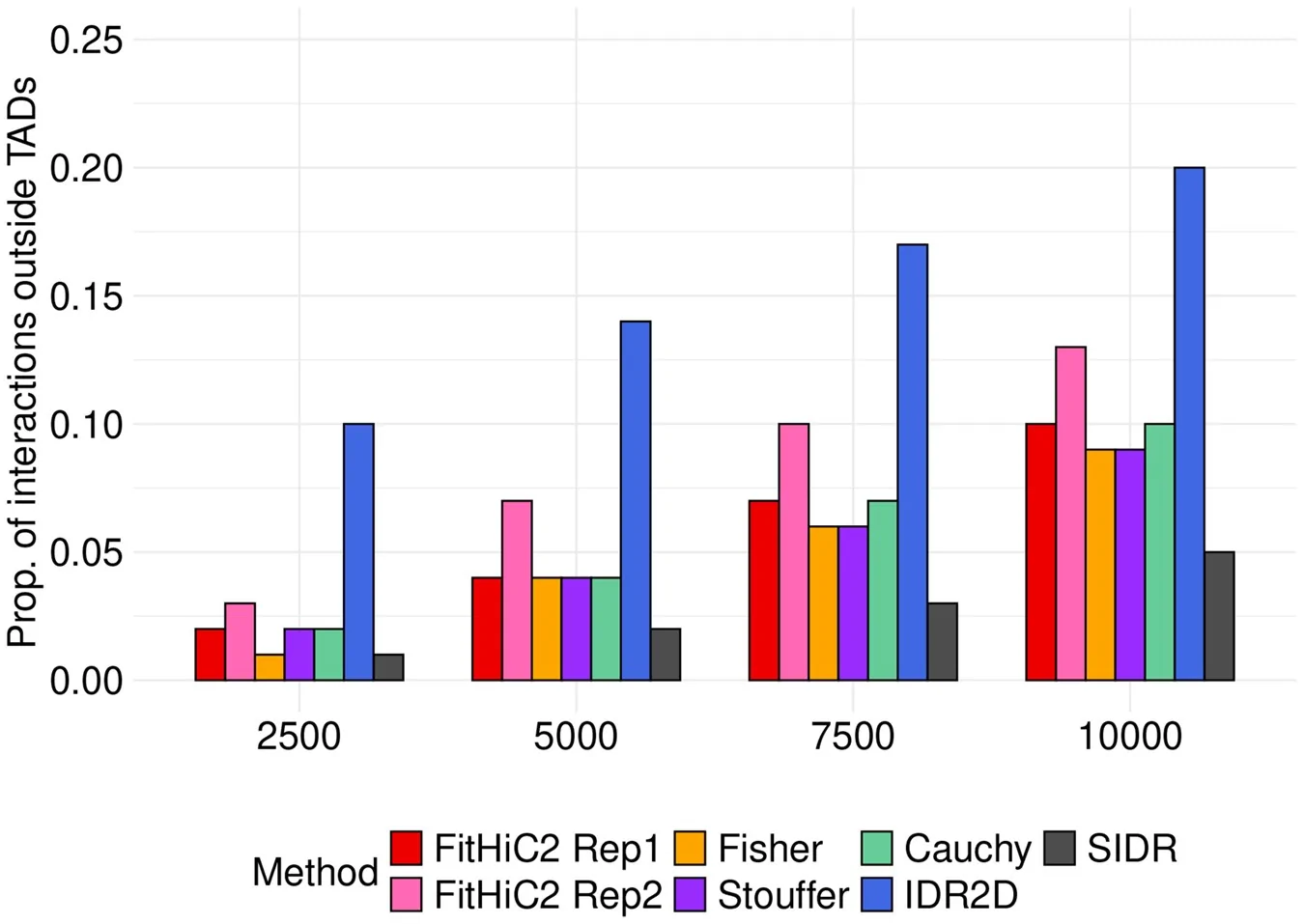
Proportions of interactions residing outside TADs among the top 2500, 5000, 7500, and 10 000 Hi-C interactions identified by FitHiC2, Fisher’s method, Stouffer’s method, the Cauchy combination test, IDR2D, and SIDR, for chromosome 18 of the mESC Hi-C data at 50 kb resolution.

To examine whether SIDR preferentially identifies such interactions, we annotated TAD structures in mESCs using OnTAD (An *et al.* 2019), which classifies interactions as outside TADs (level 0), within outer TADs (level 1), or within subTADs (level >1). We then calculated the proportion of the top n=2500, 5000, 7500, 10 000 interactions identified by each method that fall outside TADs. As shown in Fig. 7, SIDR yielded the lowest proportion of out-of-TAD interactions, suggesting stronger alignment with domain-level chromatin organization than the competing methods.

Because shorter-range interactions are more likely to lie within TADs, we further performed a distance-stratified TAD analysis to control for possible confounding by genomic distance (Supplementary Materials Section 5.2). As shown in Supplementary Fig. S6, SIDR achieves a higher proportion of within-TAD interactions across all distance strata. These results suggest that the improved TAD enrichment of SIDR is not simply a consequence of favoring shorter-range interactions, but reflects improved biological specificity after accounting for genomic distance. Consistent results were also observed in the HiC-DC+-based TAD analyses (Supplementary Figs. S9 and S10).

## 4 Discussion

We present SIDR, a stratified extension of the IDR framework, designed to quantify the reproducibility of Hi-C interactions while explicitly modeling distance-dependent effects. Unlike IDR and its two-dimensional extension IDR2D, which assume a single homogeneous dependence structure across all signals, SIDR stratifies interactions by genomic distance and estimates reproducibility parameters within each stratum. This allows SIDR to capture heterogeneity in both signal strength and replicate concordance while preserving the interpretability of the original IDR framework.

The idea of using stratification to address genomic-distance effects has also been adopted in HiCRep (Yang *et al.* 2017). However, the two methods serve fundamentally different purposes. HiCRep uses stratification to compute a stratum-adjusted correlation and summarizes reproducibility at the level of the entire contact map, providing a single global similarity measure between replicates. In contrast, SIDR uses stratification within a mixture-modeling framework to estimate interaction-level irreproducibility, explicitly separating reproducible and irreproducible signals. SIDR therefore complements HiCRep by enabling direct prioritization of high-confidence interactions rather than only assessing overall concordance between datasets.

In both simulations and real Hi-C datasets, SIDR showed consistent advantages, achieving higher power and accurate calibration in synthetic benchmarks and identified chromatin interactions enriched for functional genomic features, such as CTCF, cohesin, epigenetic states, and regulatory elements, in real datasets. SIDR also demonstrated stronger overlap with orthogonal evidence, such as ChIA-PET data and TAD-level structures, suggesting enhanced biological relevance of its discoveries.

Although SIDR requires specifying the number of strata and stratum boundaries, our analyses show that results are robust across a range of settings. In practice, we recommend defining strata based on rank correlation patterns across genomic distance, which provides a data-driven balance between interpretability and empirical performance.

Taken together, SIDR provides a principled framework for assessing interaction-level reproducibility in Hi-C data, making it a valuable tool for identifying high-confidence chromatin interactions with improved statistical rigor and functional interpretability.

## Supplementary Material

btag390_Supplementary_Data

## Data Availability

The Hi-C datasets analyzed in this study are publicly available in the Gene Expression Omnibus (GEO) database under accession number GSE35156. All other validation datasets are available in the GitHub repository at https://github.com/qunhualilab/SIDR-data.
