# Supplementary material for: Assessing reproducibility of Hi-C chromatin interactions using stratum-adjusted irreproducible discovery rate
Source: Bioinformatics. 2026 Aug 19;42(8):btag390. doi: 10.1093/bioinformatics/btag390 (PMC13509955; doi:10.1093/bioinformatics/btag390)
Supplement: Supplementary file 1 [file btag390_supplementary_data.pdf]

Supplementary Materials

1 Extension of SIDR

The SIDR framework can be extended to cases with more than two replicates. For Hi-C data with m replicates, the dependence structure can be modeled through latent variables $\mathbf{Z}_j^k = (Z_{j,1}^k, Z_{j,2}^k, \ldots, Z_{j,m}^k)$, which follow a stratum-specific multivariate Gaussian mixture distribution:

$$(Z_{j,1}^k, Z_{j,2}^k, \ldots, Z_{j,m}^k)^T \sim (1-\pi_k)\,\mathcal{N}(\mathbf{0}_m, \Sigma_0) + \pi_k\,\mathcal{N}\Big(\mu_k \mathbf{1}_m, \Sigma_1^k\Big),$$

where $\mathbf{0}_m$ and $\mathbf{1}_m$ denote the $m \times 1$ vectors of zeros and ones, respectively, and

$$\Sigma_0 = \begin{pmatrix} 1 & \rho_0 & \cdots & \rho_0 \\ \rho_0 & 1 & \cdots & \rho_0 \\ \vdots & \vdots & \ddots & \vdots \\ \rho_0 & \rho_0 & \cdots & 1 \end{pmatrix}_{m\times m}, \qquad \Sigma_1^k = \begin{pmatrix} \sigma^2 & \rho_1^k\sigma^2 & \cdots & \rho_1^k\sigma^2 \\ \rho_1^k\sigma^2 & \sigma^2 & \cdots & \rho_1^k\sigma^2 \\ \vdots & \vdots & \ddots & \vdots \\ \rho_1^k\sigma^2 & \rho_1^k\sigma^2 & \cdots & \sigma^2 \end{pmatrix}_{m\times m}.$$

Under this formulation, the irreproducible component is characterized by a common correlation ρ_0, while the reproducible component has mean $\mu_k \mathbf{1}_m$ and a stronger within-stratum correlation ρ_1^k. The estimation algorithm described in the main text can be directly used in this model.

2 Parameter Estimation

When implementing the Nelder-Mead method to maximize the log-likelihood function, we first transform the parameters to convert the constrained optimization problem into an unconstrained one. Specifically, we apply a logit transformation to both the proportion and the correlation coefficient of reproducible interactions. To guarantee that the correlation coefficient in the reproducible group ρ_1^k exceeds that in the irreproducible group ρ_0, we introduce auxiliary parameters ω_k and reparameterize ρ_0 and ρ_1^k as

$$\rho_0 = \frac{\mathrm{e}^{\mathrm{logit}(\rho_0)}}{1+\mathrm{e}^{\mathrm{logit}(\rho_0)}}, \quad \rho_1^k = \frac{\mathrm{e}^{\mathrm{logit}(\rho_0)+\omega_k^2+0.0001}}{1+\mathrm{e}^{\mathrm{logit}(\rho_0)+\omega_k^2+0.0001}}, \quad \omega_k \in \mathcal{R}$$

Since the logistic function is monotone increasing, the condition $\rho_1^k > \rho_0$ is satisfied by adding a positive quantity (the sum of the non-negative ω_k^2 and a small constant 0.0001). Accordingly, our model is reparameterized as $\boldsymbol{\theta}^* = (\pi_k, \mu_k, \rho_0, \omega_k, \sigma)$.

3 Stratification, Selection of K, and Initialization

3.1 Stratification

For a given number of strata K, we partition interactions into K strata based on the rank correlation structure across genomic distance. Specifically, we compute the rank correlation between replicates at each genomic distance, divide the resulting range into K equal-length intervals, and assign each interaction to a stratum according to the interval corresponding to its genomic distance. This yields strata that capture the empirical relationship between genomic distance and replicate dependence.

3.2 Selection of K

The choice of the number of strata K is intended to reflect the extent to which replicate dependence changes with genomic distance. We increase the number of strata only while adjacent distance ranges still exhibit meaningfully different dependence structures; once these differences become small, further stratification is unlikely to improve the model and may instead add unnecessary complexity.

In our implementation, we use a simple data-driven procedure based on rank correlations across genomic distance. Starting from $K = 2$, we evaluate candidate values of K sequentially. For each K, interactions are partitioned into K distance-defined strata using the stratification procedure described in Section 3.1, the rank correlation between replicates is computed within each stratum, and the absolute differences in correlation between adjacent strata are examined. We stop increasing K when these adjacent differences are no longer all greater than or equal to a pre-specified threshold (0.1 in our implementation). The selected K is the largest value before this condition fails.

3.3 Initialization

We specify the initial parameter values used for numerical optimization.

For the mixture proportion of the reproducible component, we impose a monotonicity constraint such that it decreases with increasing genomic distance. Specifically, for K strata, the initial values of the mixture proportions are set as

$$\frac{K}{K+1}, \frac{K-1}{K+1}, \dots, \frac{1}{K+1},$$

which reflects the expectation that shorter-distance interactions are more likely to be reproducible.

For the mean of the reproducible component, we examine a grid of candidate values and find that initializing the means at values around 1.5 yields good performance in practice. Accordingly, we set the initial values as

$$1.5,\ 1.5 - 0.1,\ \dots,\ 1.5 - 0.1 \times (K - 1),$$

which reflects the expected decrease in signal strength with increasing genomic distance.

For the standard deviation of the reproducible component, we set the initial value to 1, same as that used in the IDR model.

For the correlation coefficient of the reproducible component, within each stratum, we estimate it empirically based on the rank correlation of Hi-C signals, using the top 30% of interactions ranked in both replicates.

For the correlation coefficient of the irreproducible component, we constrain it to be smaller than the empirical correlation estimated from the top 30% of interactions in the stratum corresponding to the longest genomic distances. By default, this parameter is set to 0.15, although users may adjust it based on their specific datasets.

4 Simulation Results

In the simulation study, we considered four settings: three with distance-dependent structure ($K = 3, 4, 5$) and one without distance dependence ($K = 1$). Each setting includes 100 simulated datasets.

4.1 With Distance Dependence

In the distance-dependent settings, each dataset contains 10 000 pairs of z-statistics generated from a two-component Gaussian mixture model. Specifically, for stratum k, we generated

$$\begin{pmatrix} z_{j,1}^k \\ z_{j,2}^k \end{pmatrix} \sim (1-\pi_k)\mathcal{N}\left(\begin{pmatrix} 0 \\ 0 \end{pmatrix}, \begin{pmatrix} 1 & \rho_0 \\ \rho_0 & 1 \end{pmatrix} \right) + \pi_k \mathcal{N}\left(\begin{pmatrix} \mu_k \\ \mu_k \end{pmatrix}, \begin{pmatrix} \sigma^2 & \rho_1^k(\omega_k)\sigma^2 \\ \rho_1^k(\omega_k)\sigma^2 & \sigma^2 \end{pmatrix} \right),\ j = 1, \ldots, n_k,\ k = 1, \ldots, K,$$

where n_k is the number of observations in stratum k. The simulation parameters and their estimates under $K = 4$ are summarized in Supplementary Table S1.

Supplementary Table S1: True parameters and estimated parameters under 100 simulated datasets when $K = 4$.

		π_1	π_2	π_3	π_4	μ_1	μ_2	μ_3	μ_4
$\boldsymbol{K=4}$	True Parameters	0.80	0.55	0.30	0.05	1.10	0.80	1.20	1.30
	Estimated Values	0.78	0.54	0.32	0.06	1.16	0.80	1.16	1.28
	Standard Error	0.06	0.08	0.07	0.04	0.19	0.15	0.19	0.34
		ρ_0	ω_1	ω_2	ω_3	ω_4	σ		
	True Parameters	0.15	1.35	1.12	0.85	0.76	1.30		
	Estimated Values	0.15	1.34	1.13	0.88	0.74	1.33		
	Standard Error	0.02	0.07	0.11	0.23	0.46	0.10		

For $K = 3$ and $K = 5$, the distance strata were defined as follows:

$$\boldsymbol{K=3} : D_1 = (0, 1.62],\ D_2 = (1.62, 3.89],\ D_3 = (3.89, +\infty);$$

$$\boldsymbol{K=5} : D_1 = (0, 0.61],\ D_2 = (0.61, 1.87],\ D_3 = (1.87, 2.98],\ D_4 = (2.98, 5.04],\ D_5 = (5.04, +\infty).$$

Following the SIDR estimation procedure, the true and estimated parameters are summarized in Supplementary Tables S2 and S3.

Supplementary Table S2: True parameters and estimated parameters under 100 simulated datasets when $K = 3$.

		π_1	π_2	π_3	μ_1	μ_2	μ_3	ρ_0	ω_1	ω_2	ω_3	σ
$\boldsymbol{K = 3}$	True Parameters	0.80	0.40	0.20	1.10	1.00	0.90	0.20	1.16	0.75	0.56	1.00
	Estimated Values	0.81	0.44	0.23	1.09	0.96	0.85	0.21	1.15	0.68	0.49	1.02
	Standard Error	0.07	0.10	0.10	0.17	0.18	0.19	0.03	0.09	0.36	0.43	0.09

Supplementary Table S3: True parameters and estimated parameters under 100 simulated datasets when $K = 5$.

		π_1	π_2	π_3	π_4	π_5	μ_1	μ_2	μ_3	μ_4	μ_5
$\boldsymbol{K = 5}$	True Parameters	0.85	0.50	0.30	0.15	0.05	1.20	1.10	0.80	0.90	1.00
	Estimated Values	0.84	0.53	0.35	0.17	0.06	1.22	1.09	0.76	0.90	1.03
	Standard Error	0.06	0.08	0.14	0.09	0.06	0.24	0.16	0.20	0.24	0.37
		ρ_0	ω_1	ω_2	ω_3	ω_4	ω_5	σ			
	True Parameters	0.15	1.41	1.16	0.95	0.98	0.69	1.00			
	Estimated Values	0.15	1.40	1.16	0.94	0.92	0.59	1.04			
	Standard Error	0.01	0.06	0.11	0.29	0.44	0.64	0.10			

We further compared SIDR with IDR2D and other classical P-value combination methods in terms of discriminative power and calibration for $K = 3$ and $K = 5$. As illustrated in Supplementary Figs. S1 and S2, consistent with the results observed for $K = 4$, SIDR exhibited improved discriminative power while maintaining satisfactory calibration.

4.2 Without Distance Dependence

In the setting without distance dependence ($K = 1$), each dataset consists of 10 000 pairs of z-statistics, $(z_{j,1}, z_{j,2})^T$, $j = 1, \ldots, 10\,000$, from the mixture distribution

$$\begin{pmatrix} z_{j,1} \\ z_{j,2} \end{pmatrix} \sim 0.5\,\mathcal{N}\left(\begin{pmatrix} 0 \\ 0 \end{pmatrix}, \begin{pmatrix} 1 & 0.15 \\ 0.15 & 1 \end{pmatrix}\right) + 0.5\,\mathcal{N}\left(\begin{pmatrix} 1 \\ 1 \end{pmatrix}, \begin{pmatrix} 1 & 0.32 \\ 0.32 & 1 \end{pmatrix}\right).$$

Each generated z-statistic was converted to a one-sided P-value by testing $H_0 : \mu = 0$ versus $H_1 : \mu > 0$ via $p_{j,l} = 1 - \Phi(z_{j,l})$, where Φ denotes the standard normal CDF. The resulting P-values were used as significance scores for each bin pair. Because all observations are drawn from the same Gaussian mixture, this setting serves as a baseline for assessing performance in the absence of distance-dependent structure.

We evaluated SIDR with three choices for the number of strata ($K = 3, 4, 5$) and compared it with IDR2D and the standard P-value combination methods under this setting. As shown in Supplementary Fig. S3, SIDR has similar ROC as the non-stratified alternatives, when stratification was unnecessary.

These results suggest that SIDR does not meaningfully overfit performance in the absence of distance dependence, while still providing clear advantages when such structure is present.

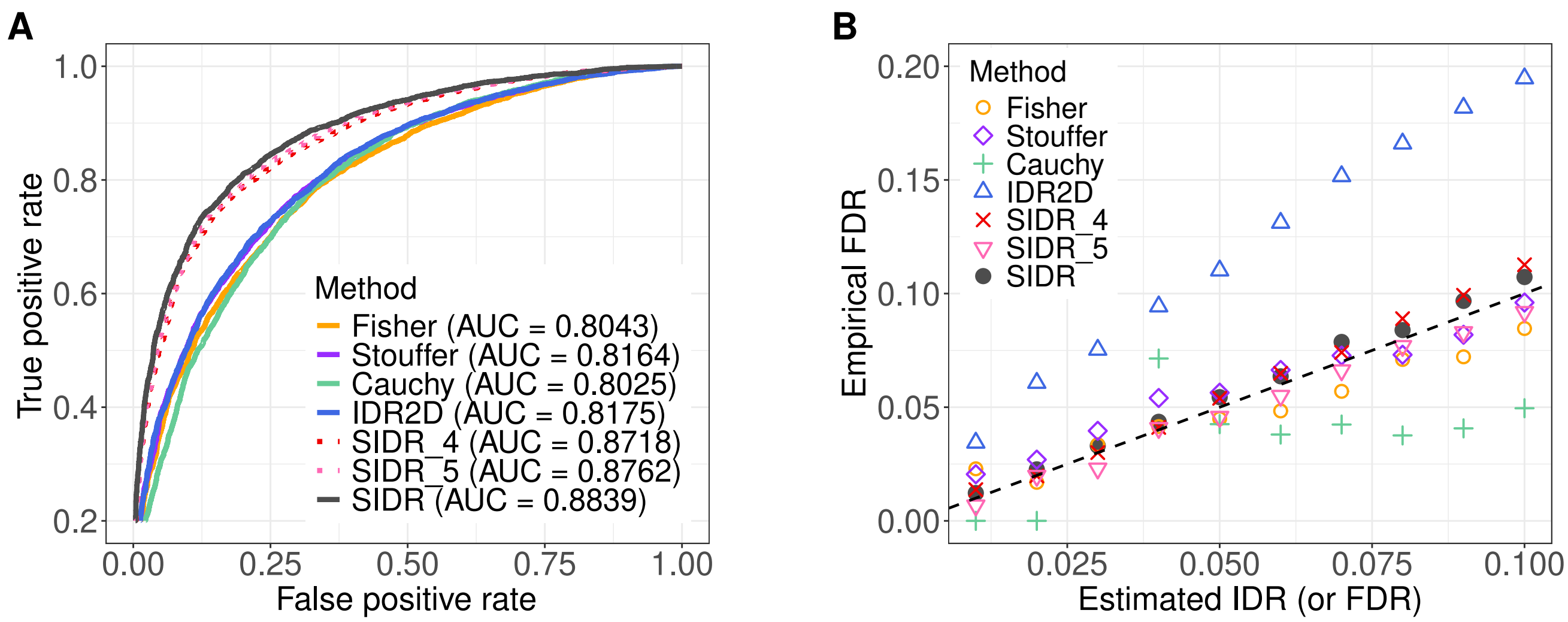

Supplementary Fig. S1: Comparison of Fisher's method, Stouffer's method, the Cauchy combination test, IDR2D, and SIDR using the true number of strata ($K = 3$), as well as SIDR (SIDR_4 and SIDR_5) with alternative numbers of strata ($K = 4$ and $K = 5$). Results are evaluated based on: **A.** The proportion of true positives versus the proportion of false positives; **B.** The estimated error rate versus the empirical error rate.

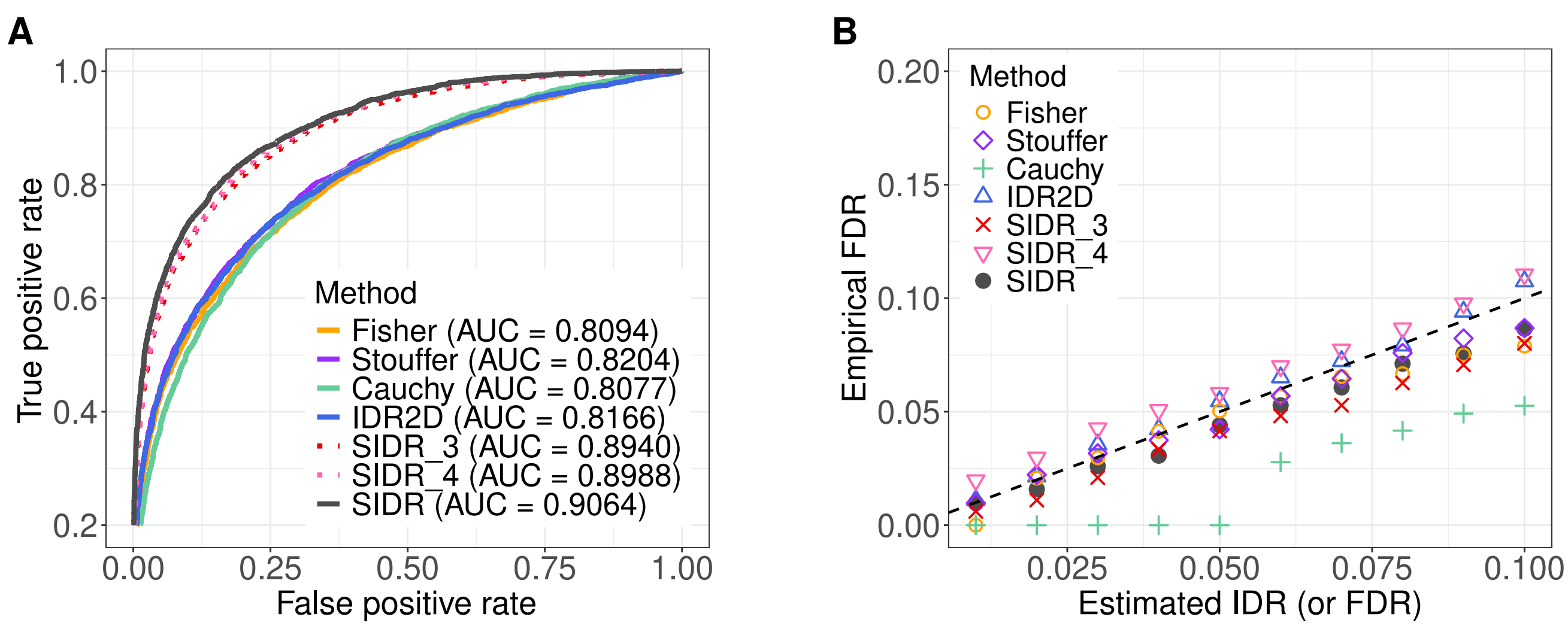

Supplementary Fig. S2: Comparison of Fisher's method, Stouffer's method, the Cauchy combination test, IDR2D, and SIDR using the true number of strata ($K = 5$), as well as SIDR (SIDR_3 and SIDR_4) with alternative numbers of strata ($K = 3$ and $K = 4$). Results are evaluated based on: **A.** The proportion of true positives versus the proportion of false positives; **B.** The estimated error rate versus the empirical error rate.

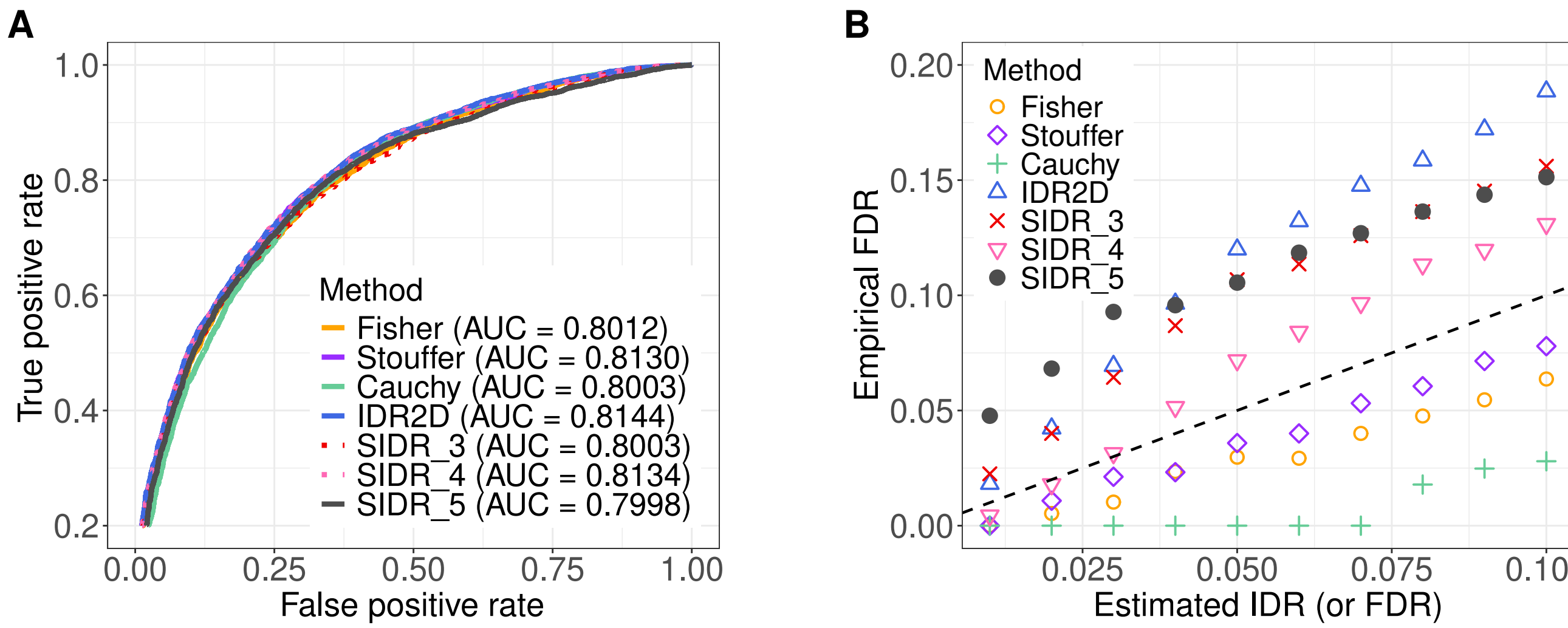

Supplementary Fig. S3: Performance comparison under a simulation setting without distance dependence. Fisher's method, Stouffer's method, the Cauchy combination test, IDR2D, and SIDR with different numbers of strata ($K = 3, 4, 5$) are compared. **A.** True positive rate versus false positive rate; **B.** Estimated error rate versus empirical error rate.

5 Real Data Analysis

In the real data analysis, we applied SIDR to chromosome 18 of both IMR90 and mESC Hi-C data at 50 kb resolution. Only chromatin interactions with genomic distances within 3 Mb were considered.

5.1 IMR90 Hi-C Results

Based on the FitHiC2 scores for IMR90 Hi-C data, we compared SIDR against individual replicates, Fisher's method, Stouffer's method, the Cauchy combination test, and IDR2D. We first evaluated the enrichment of CTCF and cohesin-binding sites. As shown in Supplementary Fig. S4, enrichment decreased as the number of top-ranked interactions n increased, demonstrating the positive correlation between CTCF (RAD21, SMC3) binding and Hi-C interactions. Overall, SIDR consistently achieved higher enrichment than competing methods.

We also explored the enrichment of epigenetic states, with the results summarized in Supplementary Fig. S5. As shown in Supplementary Fig. S5, SIDR achieved the strongest enrichment for DNase, active enhancers, and weak enhancers, and ranked a close second to IDR2D for the remaining ChromHMM states. This pattern suggests that SIDR is particularly effective at capturing regulatory interactions anchored at enhancers and open chromatin regions, which are central to long-range gene regulation. The ability to prioritize enhancer- and DNase-associated interactions highlights SIDR's strength in identifying chromatin loops likely to involve distal regulatory elements, while still maintaining competitive performance for promoter- and transcription-associated states.

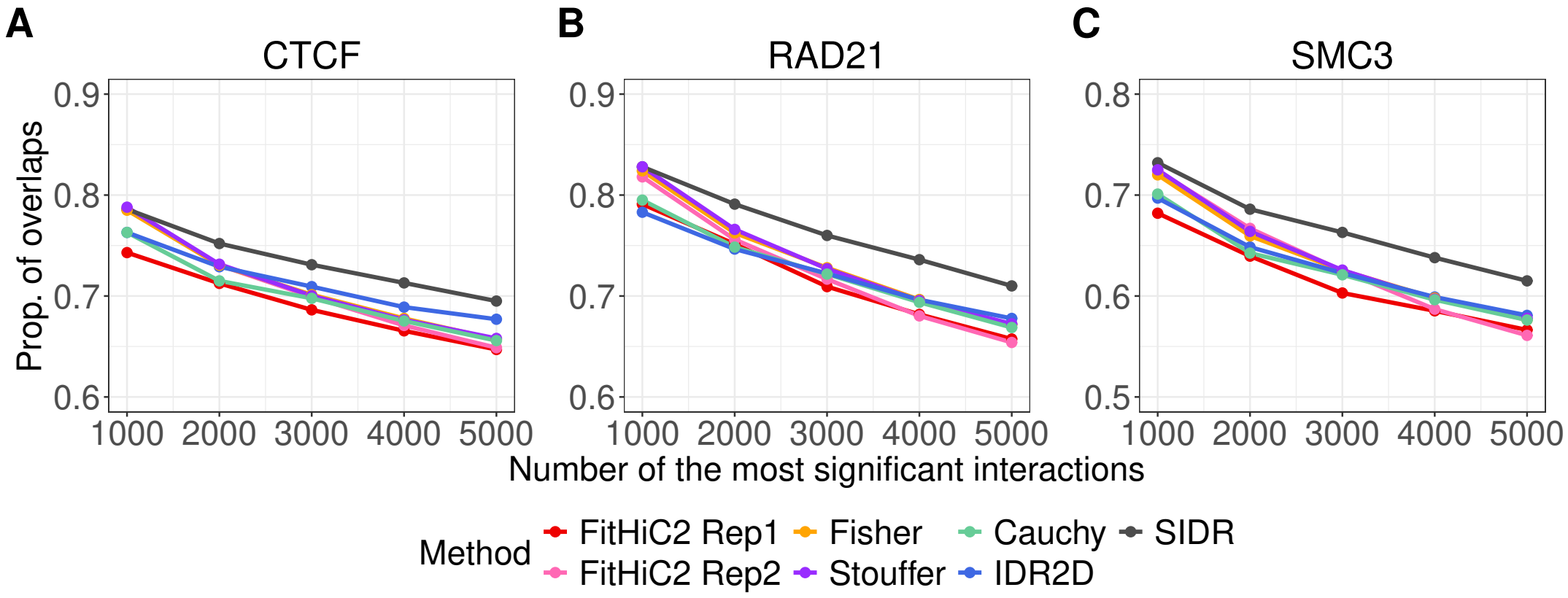

Supplementary Fig. S4: Proportions of the top 1000, 2000, 3000, 4000, and 5000 interactions identified by FitHiC2, Fisher's method, Stouffer's method, the Cauchy combination test, IDR2D, and SIDR, which overlap **A.** CTCF signals, **B.** RAD21 signals, and **C.** SMC3 signals, for chromosome 18 of IMR90 Hi-C data at 50 kb resolution.

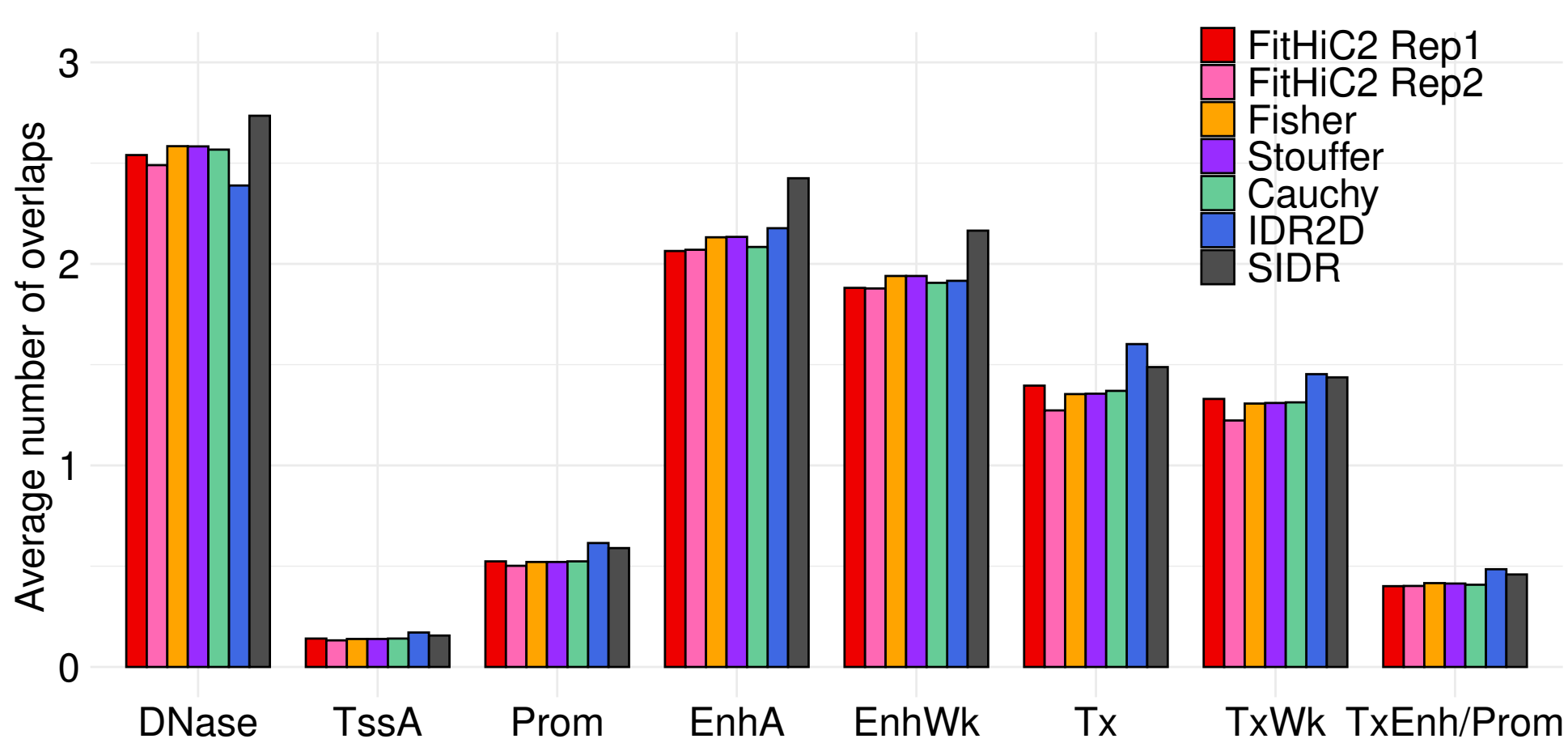

Supplementary Fig. S5: Average numbers of epigenetic states overlapping the top 5000 interactions identified by FitHiC2, Fisher's method, Stouffer's method, the Cauchy combination test, IDR2D, and SIDR, for chromosome 18 of IMR90 Hi-C data at 50 kb resolution.

5.2 mESC Hi-C Results

To assess whether SIDR preferentially identifies interactions within TADs across genomic distances, we conducted a distance-stratified TAD analysis. For each method, we selected the top 5000 interactions and computed the proportion falling within TADs for each genomic distance stratum. For the mESC data, we followed the same five-stratum partition used in SIDR; due to sparse data at the longest distances, we merged the two longest-distance strata.

As shown in Supplementary Fig. S6, SIDR achieves a higher proportion of within-TAD interactions across all strata.

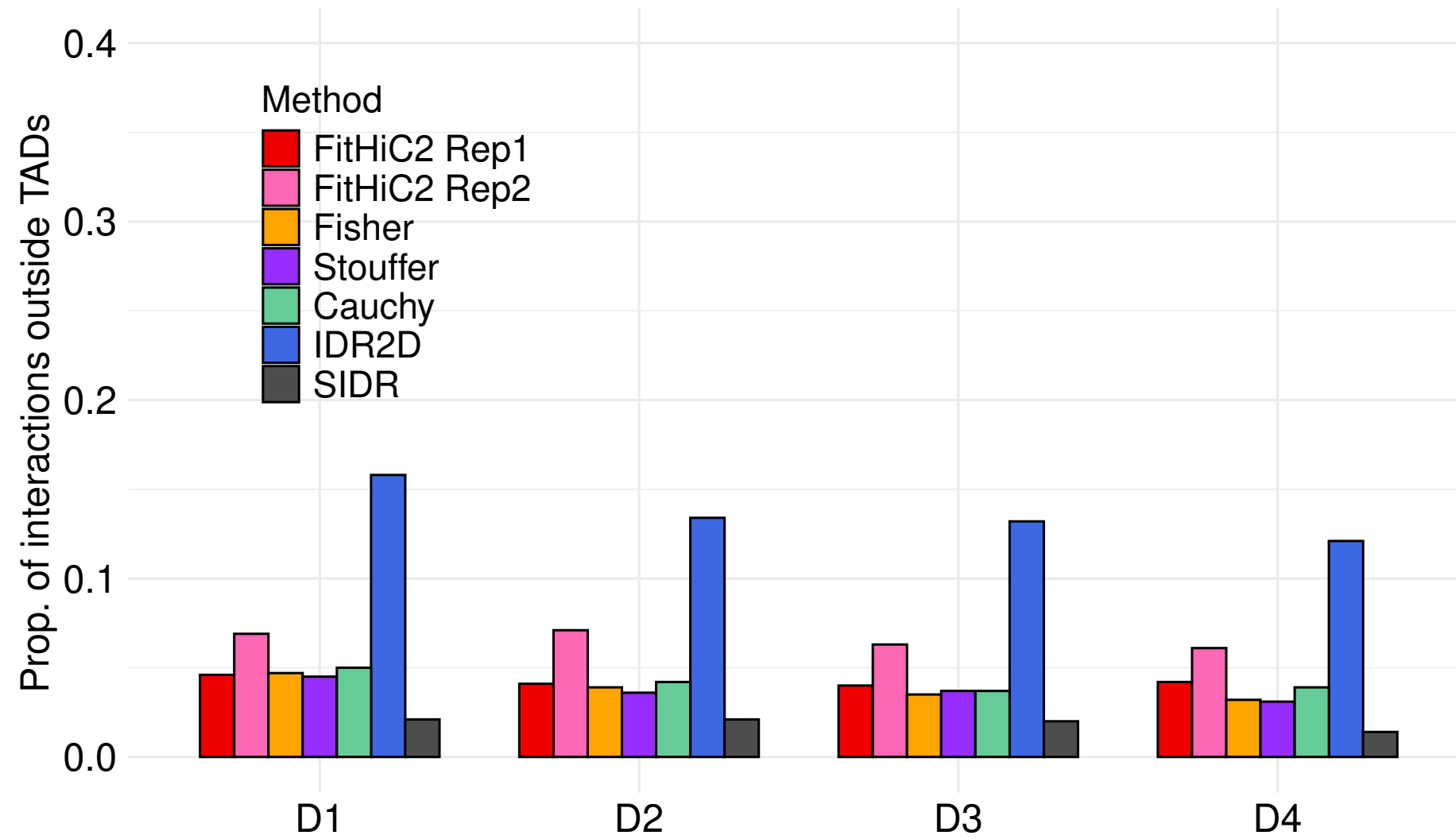

Supplementary Fig. S6: Proportions of outside-TAD interactions among the top 5000 Hi-C interactions identified by FitHiC2, Fisher's method, Stouffer's method, the Cauchy combination test, IDR, and SIDR, stratified by genomic distance for chromosome 18 of mESC Hi-C data at 50 kb resolution: D1 (0, 700] kb; D2 (700, 1500] kb; D3 (1500, 1900] kb; and D4 (1900, 3000] kb. The two longest-distance strata in SIDR were combined into D4 for visualization because they contained few interactions.

We applied HiC-DC+ to mESC Hi-C data. After excluding bin pairs with zero genomic distance, we combined the resulting P-values using Fisher's method, Stouffer's method, the Cauchy combination test, IDR2D, and SIDR, and performed the same comparative analyses as for FitHiC2 P-values.

In the ChIA-PET validation, Supplementary Fig. S7A showed that HiC-DC+ performed substantially worse than FitHiC2, capturing only 6–13% of RNAPII-mediated chromatin interactions compared to 63–76% for FitHiC2. The ROC curve (Supplementary Fig. S7B) further indicated that HiC-DC+ had limited ability to distinguish RNAPII-mediated interacting bin pairs from non-interacting ones. By contrast, Fisher's method, Stouffer's method, and the Cauchy combination test did not improve the detection of RNAPII-mediated interactions, whereas both IDR2D and SIDR increased their proportion and enhanced discriminative power. Notably, SIDR captured more than three times as many RNAPII-mediated interactions as HiC-DC+ and achieved over a 30% increase in AUC.

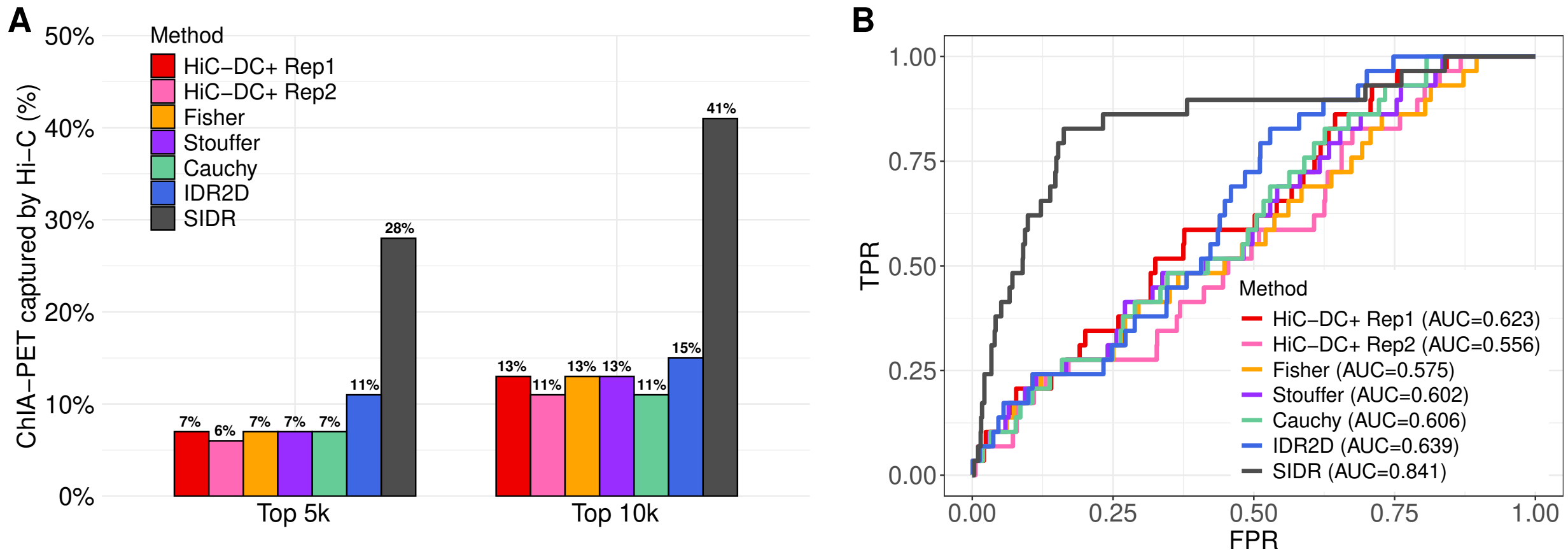

Supplementary Fig. S7: Validation of mESC ChIA-PET data. For chromosome 18 of 50kb resolution mESC Hi-C data, **A.** percentages of RNAPII ChIA-PET interactions captured by the top 5000 and 10 000 Hi-C interactions identified by HiC-DC+, Fisher's method, Stouffer's method, the Cauchy combination test, IDR2D, and SIDR. **B.** ROC curves for identifying true interactions annotated by both RNAPII ChIA-PET data and RNAPII ChIP-seq data.

When identifying CTCF-CTCF, enhancer-enhancer, enhancer-promoter, and promoter-promoter interactions, HiC-DC+ performed slightly worse than FitHiC2, showing lower percentages across all interaction types. Consistent with the FitHiC2 results, all combination methods except SIDR showed little improvement in recovering these interactions (Supplementary Fig. S8).

Furthermore, SIDR yields the lowest proportion of out-of-TAD interactions (Supplementary Fig. S9), consistent with the FitHiC2-based results. The distance-stratified analysis similarly shows that SIDR achieves a higher within-TAD proportion in each distance stratum (Supplementary Fig. S10).

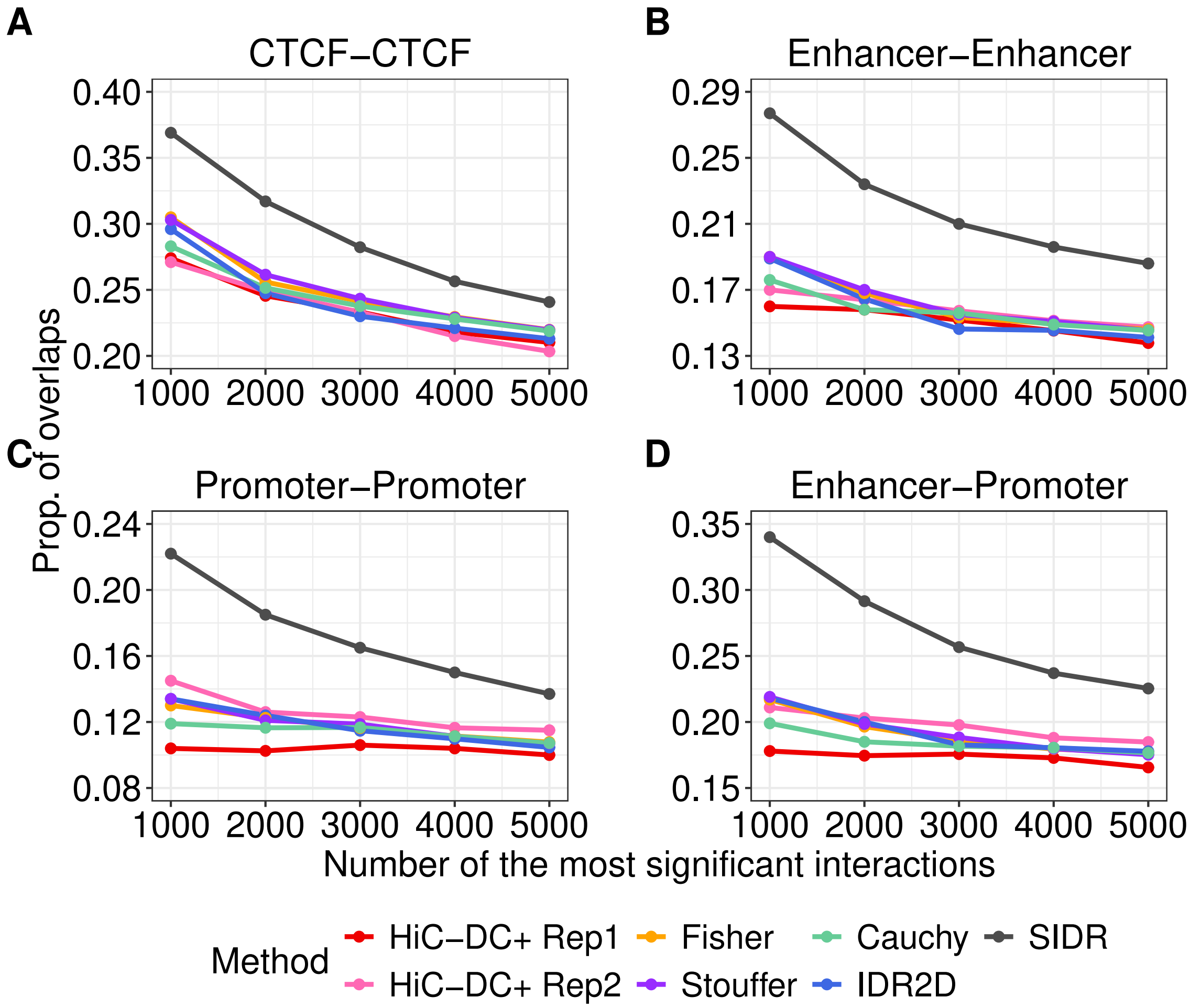

Supplementary Fig. S8: Percentages of **A.** CTCF-CTCF, **B.** Enhancer-Enhancer, **C.** Promoter-Promoter, and **D.** Enhancer-Promoter interactions among the top 1000, 2000, 3000, 4000, and 5000 interactions identified by HiC-DC+, Fisher's method, Stouffer's method, the Cauchy combination test, IDR2D, and SIDR, for chromosome 18 of mESC Hi-C data at 50 kb resolution.

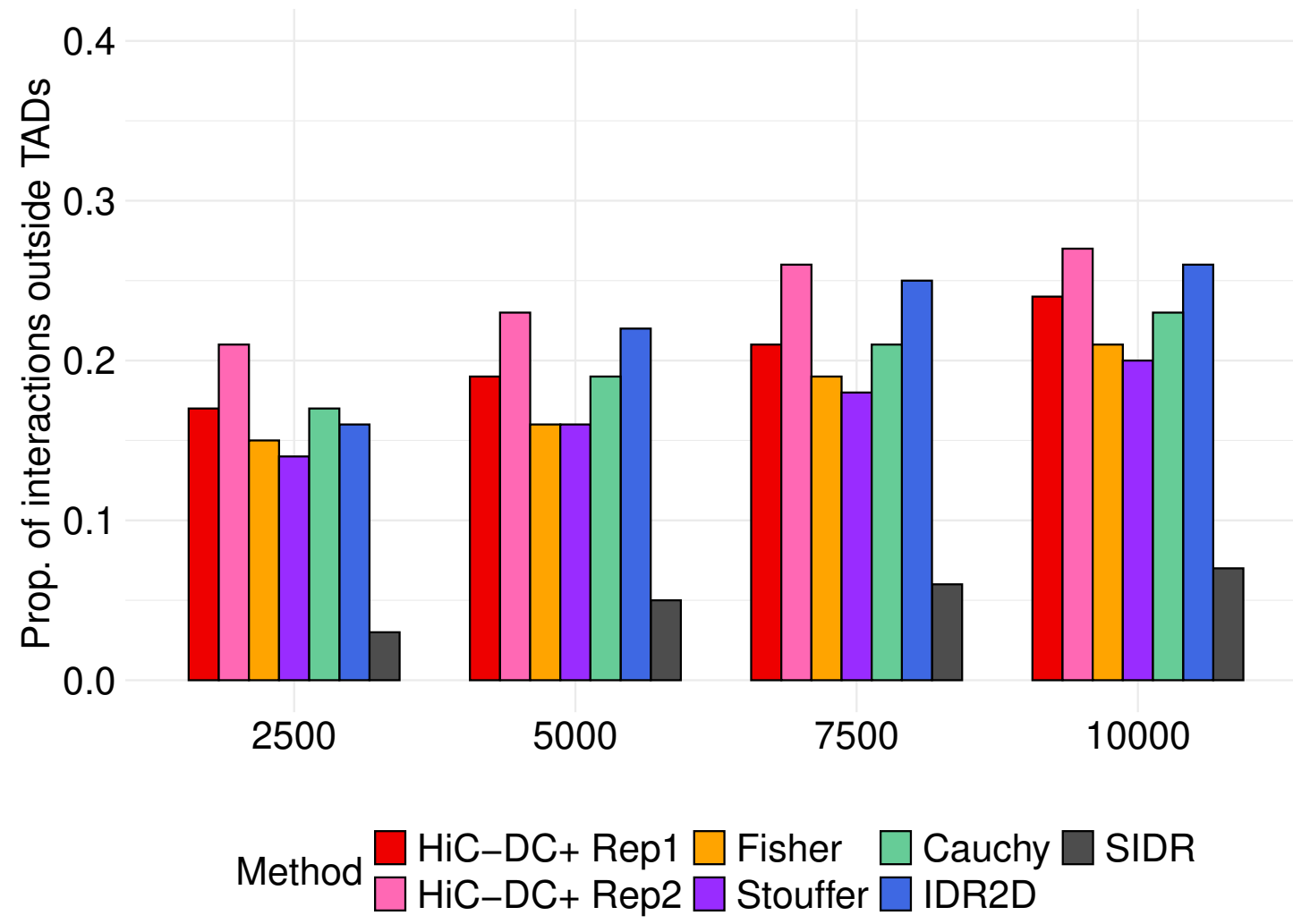

Supplementary Fig. S9: Proportions of interactions residing outside TADs among the top 2500, 5000, 7500, and 10 000 Hi-C interactions identified by HiC-DC+, Fisher's method, Stouffer's method, the Cauchy combination test, IDR2D, and SIDR, for chromosome 18 of the mESC Hi-C data at 50 kb resolution.

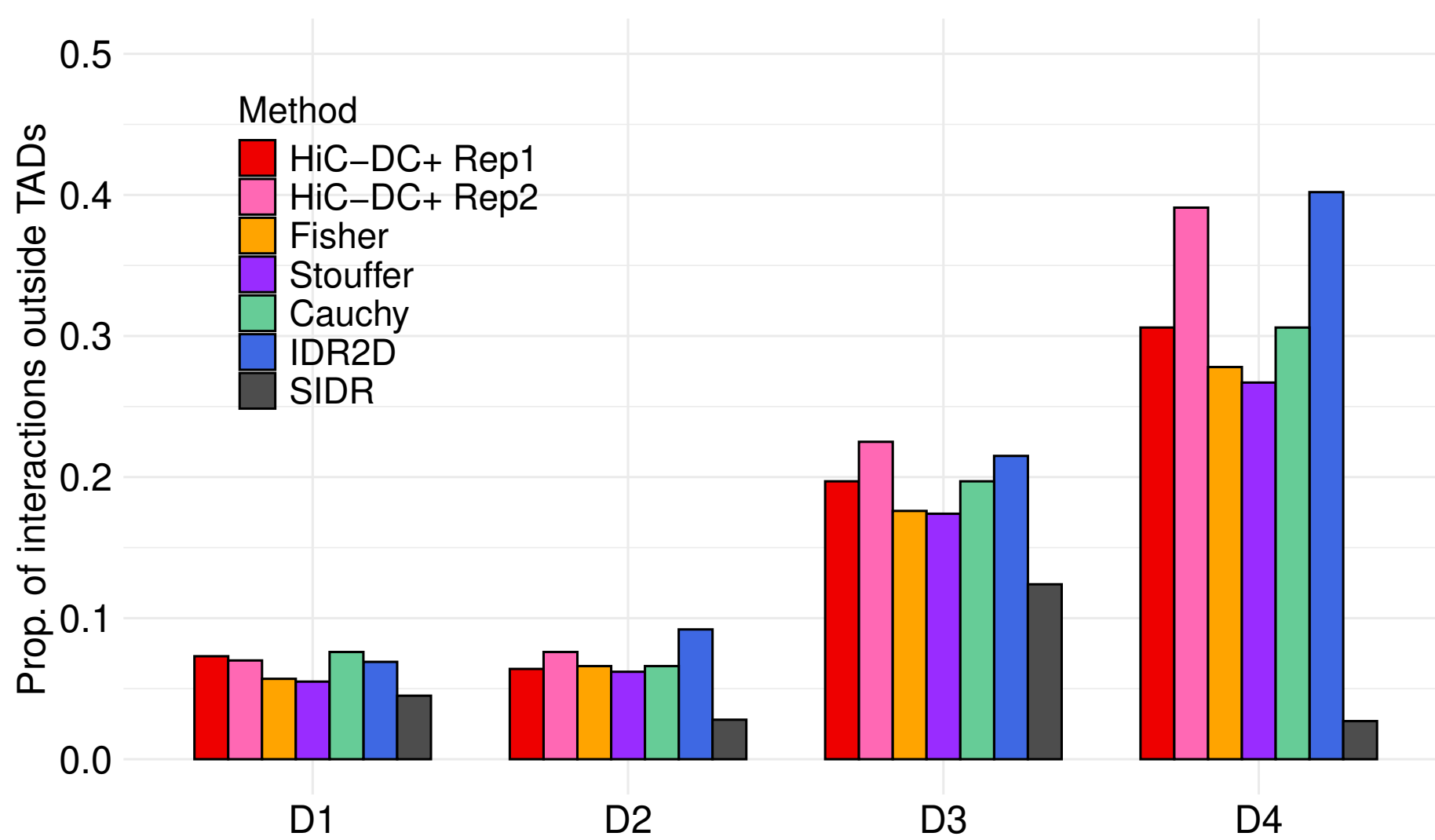

Supplementary Fig. S10: Proportions of outside-TAD interactions among the top 5000 Hi-C interactions identified by HiC-DC+, Fisher's method, Stouffer's method, the Cauchy combination test, IDR2D, and SIDR, stratified by genomic distance for chromosome 18 of mESC Hi-C data at 50 kb resolution: D1 (0-350] kb, D2 (350-1200] kb, D3 (1200-1650] kb, and D4 (1650-3000] kb. The two longest-distance strata in SIDR were combined into D4 for visualization because they contained few interactions.

6 Data Availability

For the real data analysis, IMR90 was mapped to the hg19 reference genome and mESC to the mm9 reference genome. All data sources are summarized in Supplementary Table S4.

Supplementary Table S4: Data sources used in SIDR analyses.

Data	Source
IMR90 Hi-C Rep1	GSE35156_GSM862724
IMR90 Hi-C Rep2	GSE35156_GSM892307
mESC Hi-C Rep1	GSE35156_GSM862720
mESC Hi-C Rep2	GSE35156_GSM862721
IMR90 CTCF ChIP-seq	ENCFF453XKM.bed
IMR90 RAD21 ChIP-seq	ENCFF195CYT.bed
IMR90 SMC3 ChIP-seq	ENCFF116RLU.bed
mESC CTCF ChIP-seq	mESC_CTCF.bed
mESC RNAPII ChIP-seq	mesc_RNAPII_ChIP.csv
IMR90 Epigenetic states	E017_25_imputed12marks_dense.bed.zip
mESC Epigenetic states	mESC_cStates_HMM.zip
mESC RNAPII ChIA-PET	41586_2013_BFnature12716_MOESM19_ESM.xls

Details of the Hi-C datasets used in the analysis are summarized in Supplementary Table S5.

Supplementary Table S5: Summary of Hi-C datasets.

Dataset	Chromosome	Resolution	Total counts	Replicate type
IMR90 Rep1	18	50 kb	2,128,821	Biological
IMR90 Rep2	18	50 kb	1,555,413	Biological
mESC Rep1	18	50 kb	8,791,277	Biological
mESC Rep2	18	50 kb	6,307,389	Biological

7 Runtime and Memory Usage

All analyses were performed on a machine with an Apple M4 processor and 24 GB of RAM running macOS. The runtime and memory usage of SIDR on the two Hi-C datasets are summarized in Supplementary Table S6.

Supplementary Table S6: Runtime and memory usage of SIDR on Hi-C datasets.

Dataset	Peak caller	Runtime	Memory usage
IMR90	FitHiC2	$\sim$16 min	5.8 MB
IMR90	HiC-DC+	$\sim$19 min	6.9 MB
mESC	FitHiC2	$\sim$21 min	8.9 MB
mESC	HiC-DC+	$\sim$23 min	9.6 MB
